# Alterations in Gut Microbiota and Upregulations of VPAC2 and Intestinal Tight Junctions Correlate with Anti-Inflammatory Effects of Electroacupuncture in Colitis Mice with Sleep Fragmentation

**DOI:** 10.3390/biology11070962

**Published:** 2022-06-25

**Authors:** Geng-Hao Liu, Xin-Cheng Zhuo, Yueh-Hsiang Huang, Hsuan-Miao Liu, Ren-Chin Wu, Chia-Jung Kuo, Ning-Hung Chen, Li-Pang Chuang, Shih-Wei Lin, Yen-Lung Chen, Huang-Yu Yang, Tzung-Yan Lee

**Affiliations:** 1 School of Traditional Chinese Medicine, College of Medicine, Chang Gung University, Taoyuan 333323, Taiwan; ghliu.wis@gmail.com (G.-H.L.); renchin.wu@gmail.com (R.-C.W.); ninghung@yahoo.com.tw (N.-H.C.); 2 Graduate Institute of Traditional Chinese Medicine, School of Traditional Chinese Medicine, College of Medicine, Chang Gung University, Taoyuan 333323, Taiwan; miaowhale@gmail.com; 3 Division of Acupuncture and Moxibustion, Center for Traditional Chinese Medicine, Chang Gung Memorial Hospital, Taoyuan 333423, Taiwan; ruskinchen@gmail.com; 4 Sleep Center, Chang Gung Memorial Hospital, Taoyuan 333008, Taiwan; lpchuang@gmail.com (L.-P.C.); ec108146@cgmh.org.tw (S.-W.L.); 5 Department of General Medicine, Taipei Medical University Hospital, Taipei 110301, Taiwan; m0937869275@gmail.com; 6 Division of Chinese Internal Medicine, Center for Traditional Chinese Medicine, Chang Gung Memorial Hospital, Taipei 105406, Taiwan; igighuang@gmail.com; 7 Department of Anatomic Pathology, Linkou Chang Gung Memorial Hospital, Taoyuan 333423, Taiwan; 8 Department of Gastroenterology and Hepatology, Linkou Chang Gung Memorial Hospital, Taoyuan 333423, Taiwan; m7011@cgmh.org.tw; 9 Department of Pulmonary and Critical Care Medicine, Linkou Chang Gung Memorial Hospital, Taoyuan 333423, Taiwan; 10 Department of Nephrology, Linkou Chang Gung Memorial Hospital, Taoyuan 333423, Taiwan; 11 School of Medicine, College of Medicine, Chang Gung University, Taoyuan 333323, Taiwan; 12 Bloomberg School of Public Health, Johns Hopkins University, Baltimore, MD 21287, USA; 13 Department of Traditional Chinese Medicine, Chang Gung Memorial Hospital, Keelung 204201, Taiwan

**Keywords:** inflammatory bowel disease (IBD), sleep fragmentation, electroacupuncture, ST36, intestinal tight junction, gut microbiota, vasoactive intestinal peptide receptor 2 (VPAC2), innate lymphoid cells (ILC)

## Abstract

**Simple Summary:**

Along with the modernization of society and people getting older, sleep disturbances and gut health have been identified as two key factors influencing aging, with dramatic effects on immunity and metabolism. Sleep is closely related to the gut, reflects the degree of chronic inflammation, and is associated with many degenerative diseases, hence the term “inflammaging”. This article addresses how sleep fragmentation affects the inflammatory state of the gut and elucidates the effects of restorative sleep and acupuncture on inflammatory gut remodeling and gut microbial recovery. In summary, fragmented sleep disrupted intestinal repair in mice with colitis, while electroacupuncture demonstrated likely results in alleviating colon inflammation, including maintaining colon length and daily body weight changes. In addition, the structure of the microbiota changed with decreasing gut inflammatory status. The intestinal tight junction proteins may be the key mechanism of electroacupuncture in treating sleep-fragmented ulcerative colitis mice. Electroacupuncture affects VIP through VPAC2 and further regulates intestinal mucosal immunity. This experiment demonstrates how physical stimulation stabilizes the intestinal epithelium and exerts an important anti-inflammatory effect.

**Abstract:**

The relationship between inflammatory bowel disease and sleep disturbances is complicated and of increasing interest. We investigated the inflammatory and immunological consequences of EA in sleep-deprived colitis and found that dextran sulfate sodium (DSS)-induced colitis in sleep-fragmented (SF) mice was more severe than that in mice with normal sleep. This increase in the severity of colitis was accompanied by reduced body weight, shortened colon length, and deteriorated disease activity index. DSS with SF mice presented obvious diminished intestinal tight junction proteins (claudin-1 and occludin), elevated proinflammatory cytokines (CRP, IFN-γ, IL-6), lowered melatonin and adiponectin levels, downregulated vasoactive intestinal peptide (VIP) type 1 and 2 receptor (VPAC1, VPAC2) expression, and decreased diversity of gut bacteria. EA ameliorated colitis severity and preserved the performance of the epithelial tight junction proteins and VIP receptors, especially VPAC2. Meanwhile, the innate lymphoid cells-derived cytokines in both group 2 (IL-4, IL5, IL-9, IL-13) and group 3 (IL-22, GM-CSF) were elevated in mice colon tissue. Furthermore, dysbiosis was confirmed in the DSS group with and without SF, and EA could maintain the species diversity. *Firmicutes* could be restored, such as *Lachnospiraceae*, and *Proteobacteria* become rebalanced, mainly *Enterobacteriaceae*, after EA intervention. On the other hand, SF plays different roles in physiological and pathological conditions. In normal mice, interrupted sleep did not affect the expression of claudin-1 and occludin. But VPAC1, VPAC2, and gut microbiota diversity, including *Burkholderiaceae* and *Rhodococcus*, were opposite to mice in an inflamed state.

## 1. Introduction

The importance of sleep disorders in inflammatory bowel disease (IBD) has recently received increasing attention, and more and more studies are investigating the relationship between the two [1]. Studies have shown that patients with IBD often suffer from sleep quality and severe sleep disorders even if they are not in the active stage of the disease [2]; on the other hand, sleep disorders are related to the disease activity of IBD [3], which can induce intestinal inflammation. For example, when patients with Crohn’s disease (CD) suffer from sleep disorders, the risk of disease flare-up is doubled [4]. In animal experiments, sleep deprivation has been proven to increase inflammatory cytokines such as tumor necrosis factor (TNF-α), interleukin (IL)-1β, IL-6, and C-reactive protein (CRP), which are clinical indicators observed in chronic inflammation or acute exacerbation [5,6]. Therefore, a disordered sleep architecture changes the immune system and affects the disease process of IBD. Recent studies have also shown that sleep dysfunction and disruption of the circadian rhythm can lead to the upregulation of inflammatory cytokines [7,8], which not only constitute a potential predisposing factor of the disease [9] but also increase the risk of malignant tumors [7]. Conversely, IBD can affect sleep quality, forming a vicious cycle that perpetuates and worsens inflammation.

Intestinal microbes can be regarded as an internal ecosystem in mammals. This ecosystem comprises a large number of bacteria and has a wide range of influences on the host. The gut microbiota plays a vital role in food digestion, metabolism, immune regulation, and even neurocognitive performance. The above physiological processes show that the gut microbiota has diurnal characteristics [10]. In addition, intestinal epithelial cells rhythmically sense microbial changes, which are very important for the homeostasis of the intestinal epithelium [11]. On the other hand, the composition of the intestinal microbiota itself and the physiological oscillations exhibited by it also follow the host’s feeding rhythm and change periodically [10]. In recent years, it has been discovered that many human diseases, especially some chronic inflammation-related diseases, such as obesity, IBD, cancer, and neurological disorders, are closely related to an imbalance in the normal microbiota or a disturbance in the circadian rhythm [12]. In the past few years, changes in the composition and function of intestinal microbes have become an important key to the pathogenesis of IBD and have become a hot spot in this field of research [13], as well as in chronic sleep fragmentation with chronic intermittent hypoxia mice model [14]. Recent studies have pointed out that certain intestinal bacterial strains have a direct impact on IBD [15] and sleep fragmentation [16]. However, it is still unclear whether there is a common mechanism between microbial imbalance, sleep disorder, and inflammation, or whether the three regulate each other.

Currently, various integrated medical treatments are increasingly used to relieve or control IBD, including acupuncture. Even though there is limited evidence of acupuncture in IBD treatment thus far, the therapeutic potential of acupuncture for IBD has received attention in clinical [17,18] and research [19,20] fields. On the other hand, increasing evidence supports acupuncture for treating sleep disorders, such as restless legs syndrome [21,22] or insomnia [23,24]. A systematic review covering up to July 2007 found that 93% of the 30 eligible studies confirmed that acupuncture treatment could help improve many aspects of sleep quality [25]. In terms of subjective sleep performance, it can shorten the time to fall asleep, increase sleep efficiency, improve sleep length and quality, and reduce the symptoms associated with insomnia [26]. In animal experiments, stimulating rats’ Anmian points with electroacupuncture (EA) at night can specifically increase nonrapid eye movement (NREM) sleep without affecting rapid eye movement (REM) sleep, which involves the induction of cholinergic activity in nucleus tractus solitarius (NTS) [27] and the stimulation of opioid neurons, which in turn increases the concentration of β-endorphins and the participation of μ-opioid receptors [28]. Acupuncture can alleviate autonomic nervous system disorders and decrease inflammation through the parasympathetic nerve, the hypothalamic–pituitary–adrenal axis (HPA axis), and other mechanisms, but the effect of acupuncture on intestinal microbes is unknown. This article aimed to understand the neuroimmunological mechanism of EA on gut microbiota in sleep-fragmented mice with colitis.

## 2. Materials and Methods

### 2.1. Animals

Thirty healthy male 8-week-old C57BL/6J mice weighing 20–25 gm were purchased from the National Laboratory Animal Center (Taipei, Taiwan). All the mice were raised in a fixed-temperature (22 ± 2 °C) and relative-humidity (50 ± 5% RH) environment, with regular light and dark cycles (12 h/12 h, 08:00 brightening, set as Zeitgeber Time 0, ZT0) and ad libitum access to food and water.

All animal procedures were based on the *Guiding Principles for the Care and Use of Laboratory Animals* (*Guide*, NRC 2011). Under the premise of 3R, we minimized the animal number for necessary procedures. Additionally, the experiment was approved by the Institutional Animal Care and Use Committee of Chang Gung University and Chang Gung Memorial Hospital (IACUC Approval Nos. CGU106-076 and 2019040301).

### 2.2. Experimental Groups and Protocol

**Figure 1:**
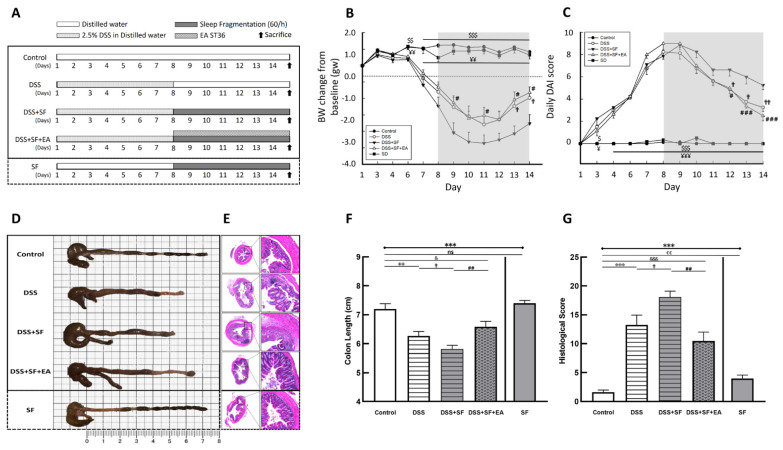
Electroacupuncture ameliorates dextran sulfate sodium (DSS)-induced colitis in sleep-fragmented C57BL/6 mice. (**A**) Experimental protocol of 2.5% DSS colitis, sleep fragmentation (SF) and electroacupuncture (EA) ST36 treatment course; (**B**) mice body weight loss after management of DSS or sleep fragmentation in five groups (statistics of differences between each group and DSS + SF group by Mann–Whitney U test); (**C**) daily DAI score in five groups (statistics of differences between each group and DSS + SF group by Mann–Whitney U test); (**D**) intestine photograph of colorectum length in each group; (**E**) representative H&E-stained colorectum sections (left: 40×, right: 200× magnification) in each group; (**F**) statistics of colon length in five groups by Kruskal–Wallis test; differences between each group by Mann–Whitney U test; (**G**) statistics of histological score in five groups by Kruskal–Wallis test; differences between each group by Mann–Whitney U test. * Stands for a result of five groups comparison (represented by bold line segments with endpoints). ✠, $, ✟, #, ¥, & and € represent the results of control group versus DSS group, control group versus DSS + SF group, DSS group versus DSS + SF group, DSS + SF group versus DSS + SF + EA group, DSS + SF group versus SF group, control group versus DSS + SF + EA group, and control group versus SF group, respectively (represented as a thin line without endpoints). $, ✟, #, ¥, &, €, *p* < 0.05; ✠✠, $$, ✟✟, ##, ¥¥, €€, *p* < 0.01; ***, ✠✠✠, $$$, ¥¥¥, &&&, *p* < 0.001; ns, no significance. Data were presented as mean ± SEM of six mice in each group. DSS, dextran sodium sulfate; SF, sleep fragmentation; EA, electroacupuncture.

We divided 24 mice into four groups, namely, the (1) control group, (2) DSS-induced colitis (DSS group), (3) DSS-induced colitis combined with sleep fragmentation (DSS + SF group), and (4) DSS-induced colitis combined with sleep fragmentation and EA ST36 (DSS + SF + EA group). In addition, we set up a simple sleep fragmentation group for comparison (SF group) [29]. The process for each group with 6 mice is shown in Figure 1A.

### 2.3. Induction of Experimental Colitis

Experimental colitis was induced by DSS (molecular weight 40,000 Da; Sigma–Aldrich, St. Louis, MO, USA) [30], which was added to the daily drinking water of mice at a concentration of 2.5% in the first seven days. The DSS water was renewed every two days.

### 2.4. Sleep Fragmentation Animal Model

After seven days of DSS induction, mice in the DSS group, DSS + SF group, DSS + SF + EA group, and SF group received sleep fragmentation via the Lafayette sleep deprivation system (#80391) [31] during the light period (ZT 0-12) from days 8 to 14. A bar was horizontally moved across the mouse cage base during the fragmented sleep period to interrupt mouse sleep. The frequency was set as interruption once per minute (60/h), similar to severe obstructive sleep apnea. Through the period of this setting motion, animals could freely drink and eat.

### 2.5. EA Intervention Procedure

Mice in the DSS + SF + EA group simultaneously received sleep fragmentation and EA from days 8 to 14. EA was performed at bilateral ST36 acupoints on both legs at ZT 10–11 (19:00–20:00) once a day. The EA process was performed under level III anesthesia and maintained by inhalation of 1.0–1.5% isoflurane. The depth of anesthesia was evaluated by pedal reflex, which meant no response to forceps touching. The mice were kept in a supine position with their limbs fully exposed. Their hind limbs were shaved and disinfected with 75% alcohol before acupuncture. The ST36 point in the mice is located 5 mm below the knee joint and 2 mm outside the front edge of the tibia [32]. Two sterilized stainless steel disposable needles (0.22 mm in diameter, 13 mm in length; purchased from Ching Ming Medical Device Co., Ltd., New Taipei, Taiwan), marked with a red marker at a distance of 2–3 mm from the tip of the needle, were inserted vertically into the muscle layer to a depth of 2–3 mm. The entire needle insertion process was completed within 15 s strictly by a single experienced acupuncturist. After the *deqi* sensation, the needle’s tail was connected to an electrical stimulator (transcutaneous electrical stimulator, MODEL-05B(6), purchased from Ching Ming Medical Device Co., Ltd., New Taipei, Taiwan), and the parameters were set to stimulate the acupoints for 15 min as a dilatational wave, 12 Hz, 1.5 Vp-p. Apparent intermittent focal muscle contractions of both lower limbs were observed during electrical stimulation.

### 2.6. Assessment of Colitis Severity

Three aspects were used to evaluate the inflammatory severity of murine colitis: (1) clinical indicators (bodyweight loss, viscosity of stool, and the severity of bloody stool); (2) macroscopic evidence of colitis (shortening of the colon length); and (3) microscopic evidence of colitis (ranking the pathological change of colon tissue).

(1) Clinical indicators

We measured the mouse’s body weight and fluid and food consumption before the rapid clinical evaluation, including stool consistency, fecal occult blood, and observation of rectal bleeding at 8:00 every morning (ZT 0-3).

Specifically, the detailed evaluation of the disease activity index (DAI) of intestinal inflammation was as follows. We weighed each animal every day to calculate the percentage of weight change compared to the first day of the experiment. If the weight loss were 1–5%, 5–10%, 10–20%, and >20%, we would give a score of 1, 2, 3, and 4, respectively. In terms of stool consistency, normal stool texture was scored as 0 points; loose, pasty, semisolid stools that did not stick to the anus were scored as 2 points; and watery stools that adhered to the anus were scored as 4 points. For the severity of rectal bleeding, a score of 0 indicated no bleeding; a score of 1 indicated a slight occult blood reaction; a score of 2 represented a positive occult blood reaction or visible pellet bleeding (Hemoccult^®^ Single Slides System, Catalog number: 60151A, purchased from the Fisher Scientific International Inc., Hampton, NH, USA); and a score of 4 indicated gross anal hemorrhage or obvious bloody stool [33]. Finally, the daily weight loss, stool consistency, and rectal bleeding severity were added together to obtain the DAI [34], which is shown in Table S1. The formula is as follows: DAI = (weight loss) + (stool consistency) + (rectal bleeding).

(2) Macroscopic evidence of colitis

On the 15th day of the experiment, the mice were sacrificed through cardiac puncture under anesthesia during 08:00–14:00 (ZT 0-6). After that, the colon tissue was removed immediately, and the colon length from the ileocecal junction to the anus was measured.

(3) Microscopic evidence of colitis

Later, a 3–5 mm specimen was removed from the proximal end of the free colon, fixed with 4% formaldehyde PBS, embedded in paraffin, and sliced into sections of 5 µm thickness for histopathological examination. Through H&E staining, the pathological changes were observed under a light microscope, and immunohistochemical evaluations were subsequently performed on these sections.

A validated scoring system was used to determine the pathological severity of colon inflammation [35,36]. In short, as shown in Table S2, according to the percentage of six characters under the fields, including goblet cell loss, mucosal thickness, inflammatory cells, submucosal infiltration, structural damage, and ulcers, the gradings were 0, 1, 2, 3, 4, representing 0, 0–25%, 25–50%, 50–75%, and 75–100%, respectively.

### 2.7. Plasma Biomarker Analysis

The animals were euthanized through intraperitoneal injection of barbiturates before cardiac puncture. After the plasmapheresis of the collected blood, the plasma was homogenized in 700 μL Tris-HCl buffer with proteinase inhibitor (Sigma-Aldrich, Corporation, Burlington, MA, USA) and centrifuged at 13,000 rpm at 4 °C for 20 min, and the supernatant was stored at −30 °C until analysis [37]. Biomarkers such as melatonin, inflammation markers (CRP, IFN-γ), IL-6, and adiponectin were assessed.

The plasma level of melatonin was measured by mouse melatonin ELISA kits (E-EL-M0788; purchased from Elabscience Biotechnology Inc., Houston, TX, USA). The lowest limit of detection was 7.81 pg/mL. The other biochemical plasma levels, including IFN-γ, IL-6, CRP, and adiponectin, were quantified with a multiplexed immunoassay system xMAP (Luminex^®^200™, Austin, TX, USA). According to the manufacturer’s instructions, IFN-γ and IL-6 were analyzed with mouse multiplex kits (IFN-γ-EPX01A-20606-901, IL-6-EPX01A-20603-901, analyzed with ProcartaPlex™ produced by Thermo Fisher Scientific Inc., Waltham, MA, USA) without dilution of the plasma; CRP was analyzed with plasma diluted 1:10,000 with ProcartaPlex™ simplex kits (EPX01A-26045-901; Thermo Fisher Scientific Inc., Waltham, MA, USA); plasma was diluted to 1:2000 when analyzing adiponectin with ProcartaPlex™ mouse simplex kits (EPX01A-26038-901; Thermo Fisher Scientific Inc., Waltham, MA, USA). The lowest limits of detection (LLOD) for quantifying IFN-γ, IL-6, CRP, and adiponectin were 1.2, 5.3, 34.0, and 584.7 pg/mL, respectively. For the statistics proposal, those values of observation lower than the lowest limits of detection (LLOD) were calculated as half of the LLOD for analysis and statistics.

### 2.8. Stool Collection, Gut Microbiota Processing, and 16S Metagenomics Analysis

The resected colon was opened longitudinally, and mouse feces were collected in a fume hood, followed by microbiota processing and analysis. The extraction of fecal DNA and sequencing of 16S rRNA were processed by Genomics BioSci & Tech. Co., Ltd. (Taipei, Taiwan). In short, 1 µL of sample DNA (10 pg~500 ng) was used as a template in the PCR amplification of the bacterial 16S rRNA variable region V3~V4, and the specific primer sets were 341F (V3_illumina 5′-CCTACGGGNGGCWGCAG-3′) and 805R (V4_illumina 5′-GACTACHVGGGTATCTAATCC-3′). The 450-500 bp fragments amplified by PCR were excised during gel extraction and purified using a QIAquick Gel Extraction Kit (QIAGEN, Hilden, Germany). The TruSeq nano DNA Library Prep Kit (Illumina, Inc., San Diego, CA, USA) was used for library construction, and the library quality was assessed on the Qubit@ 2.0 Fluorometer (Thermo Scientific, Waltham, MA, USA) and Agilent Bioanalyzer 2100 system. Finally, the library was sequenced on an Illumina MiSeq platform, generating 300 bp paired-end reads according to the manufacturer’s instructions. Afterward, the data processing was performed, and many packages were used in the metagenomics analysis workflow, including “MultiQC v1.4 (Stockholm, Sweden)”, “FastQC v0.11.5 (Cambridge, UK)”, “cutadapt v1.16 (Martin, 2011)”, and “FLASH v1.2.11 (Magoc and Salzberg, 2011)”.

Due to the vast amount of data, more than one million read pairs and paired reads were merged into amplicon sequences, and then these amplicon sequences were checked for primers, duplicates were removed, short sequences were filtered out, and the final OTU was clustered. These steps reduced the number of amplicon sequences by using the bioinformatics software packages “Mothur” v.1.39.5 (Schloss 2009 and Kozich 2013) and “QIIME” v1.9.0 (Caporaso 2010) and grouped them into representative OTUs for further analysis, including SILVA 132 ribosomal RNA (rRNA) database searches and taxonomic assignments. All valid reads were calculated for the pairwise distance between aligned DNA sequences, with a cutoff value of 0.03, and then clustered into OTUs using the “average neighbor algorithm” with a hard cutoff value of 0.03. These final OTU classifications were compared with the Greengenes 16S rRNA taxonomic database (gg_13_8), and then the taxonomy was assigned to different taxonomic levels: kingdom, phylum, class, order, family, genus, and species.

Representative sequences of OTUs and their relative abundances were used to calculate alpha diversity, including rarefaction analysis (Chao1 represents community richness, Shannon illustrates community diversity), rank abundance, and Venn diagram. Then, beta diversity (PCoA, phylogenetic tree) was used to analyze the diversity between groups.

### 2.9. Multiplex Immunoassay of Colon Tissue

The harvested proximal colon of mice was weighed and processed. Tissue extraction reagents (Invitrogen™ Tissue Extraction Reagent I, Catalog number: FNN0071, ThermoFisher Scientific, Waltham, MA, USA) with protease inhibitors (Halt™ Protease Inhibitor Cocktail, Catalog number: 87786, ThermoFisher Scientific, Waltham, MA, USA) were added to homogenize colon tissue according to the manufacturer’s guidance, followed by centrifugation at 10,000 rpm for 5 min to precipitate the tissue fragments and collect the supernatant. The Bradford protein quantification method was used to determine the protein concentration for subsequent calibration.

Multiplex immunoassays quantified 13 kinds of cytokines, including GM-CSF, IFN-γ, IL-1β, IL-4, IL-5, IL-6, IL-9, IL-10, IL-13, IL-17A, IL-22, IL-23, and TNF-α, in plasma with the xMAP Luminex^®^200™ instrument (Austin, TX, USA). According to the brand, the kits for mouse (GM-CSF-EPX01A-20612-901, IFN-γ-EPX01A-20606-901, IL-1β-EPX01A-26002-901, IL-4- EPX01A-20613-901, IL-5-EPX01A-20610-901, IL-6-EPX01A-20603-901, IL-9-EPX01A-26041-901, IL-10-EPX01A-20614-901, IL-13-EPX01A-26015-901, IL-17A-EPX01A-26001-901, IL-22-EPX01A-26022-901, IL-23-EPX01A-26017-901, TNF-α-EPX01A-20607-901, analyzed by ProcartaPlex™of Thermo Fisher, Waltham, MA, USA) analyzed GM-CSF, IFN-γ, IL-1β, IL-4, IL-5, IL-6, IL-9, IL-10, IL-13, IL-17A, IL-22, IL-23 and TNF-α without dilution, providing lowest limited detection 2.8, 0.9, 1.3, 1.4, 2.2, 4.9, 18.5, 1.9, 2.5, 1.5, 11.3, 9.7 and 3.1 pg/mL, respectively. Those values lower than the lowest limits of detection (LLOD) were regarded as half of the LLOD for analysis and statistics.

Finally, after normalizing the detected cytokine concentration (pg/mL) to the protein concentration (mg/mL), the quantitative results of each cytokine (pg/mg total protein) were obtained.

### 2.10. Immunohistochemistry Staining

To reveal the possible mechanisms of acupuncture, we used immunohistochemical staining to confirm whether the integrity of the intestinal epithelium was involved. Claudin-1 (ab15098, Abcam, Trumpington, Cambridge, UK) and occludin (CSB-PA190654, Cusabio, Houston, TX, USA) were tight junction proteins detected as a marker indicating the integrity of the barrier function. In addition, the role of the vasoactive intestinal peptide (VIP) type 1 receptor (VIPR1/VPAC1, CSB-PA052529, Cusabio, Houston, TX, USA) and type 2 receptor (VIPR2/VPAC2, A03768, Boster, Pleasanton, CA, USA) under EA were clarified by immunohistochemical staining. The slides were evaluated under 400× microscopic view. Images of IHC slides were quantified by calculating the percentage of DAB-stained area through true color image analysis with the application of adjusted thresholds in ImageJ software (NIH).

### 2.11. Western Blot

Colon tissues that had been stored at −80 °C were lysed in Thermo-Fisher Tissue Protein Extraction Reagent (Thermo Fisher Scientific, Rockford, IL, USA) containing a protease inhibitor cocktail (Roche Diagnostics, Basel, Switzerland). Protein concentrations were determined using the Bio-Rad Protein Assay (Bio-Rad, London, UK). Cell lysates (40 μg/lane) were separated on SDS–PAGE gels and transferred onto PVDF membranes (Immobilon-P, Mecrk Millipore Ltd., Darmstadt, Germany). Total extracted proteins were then incubated with tris-buffered saline containing 0.1% Tween-20 with 5% non-fat dried milk for 1 h at room temperature, and the blots were incubated with claudin-1 (ab15098, Abcam, Trumpington, Cambridge, UK), VIPR2/VPAC2 (CSB-PA060078, Cusabio, Houston, TX, USA), and β-actin (MAB1501, Millipore, Burlington, MA, USA) antibodies overnight at 4 °C. Secondary antibody was conjugated with HRP-conjugated goat anti-rabbit and anti-mouse (Dako, Santa Clara, CA, USA) secondary antibodies. Quantifications were performed using ImageJ software (NIH).

### 2.12. Statistical Analysis

Data are expressed as the mean ± standard error (mean ± SEM). We used software such as IBM SPSS Statistics 21.0 (IBM Corp., Armonk, NY), GraphPad Prism 8 (La Jolla, CA, USA), and SigmaPlot 12.0 (Systat Software Inc., San Jose, CA, USA) for analysis and explanation. Daily weight change, daily DAI, colon length, histological score, various inflammatory markers in plasma (CRP, IFN-γ), IL-6, adiponectin, melatonin, and the value of the colon tissue multifactor immunoassay results were analyzed by Kruskal–Wallis ANOVA to test whether there were significant differences between the groups. The Mann–Whitney U test was used to determine the differences between each group. If the *p*-value was less than 0.05, the difference was considered to be statistically significant.

## 3. Results

### 3.1. Severity of DSS Colitis and Intestinal Barrier Damage Was Aggravated during Sleep Fragmentation and Was Ameliorated after EA

Under the protocol of 7 days of dextran sulfate sodium (DSS) administration followed by sleep fragmentation (Figure 1A), we evaluated the inflammation status in five groups of mice by daily decreases in body weight (Figure 1B) and the index (DAI) (Figure 1C). After sacrifice, the colon length (Figure 1D,F) and the intestinal histological score (Figure 1E,G) of the mice were also measured.

Compared with mice in the control group, the body weight of the DSS group mice was significantly lower than the baseline body weight on the first day (−1.96 ± 0.34 vs. 0.84 ± 0.14 gm, *p* < 0.001), and the DAI was obviously increased (3.25 ± 0.37 vs. 0.00 ± 0.00, *p* < 0.001). The length of the colon was significantly shortened (6.27 ± 0.15 vs. 7.20 ± 0.18 cm, *p* = 0.001), and the histological inflammation score also increased significantly (13.27 ± 1.69 vs. 1.64 ± 0.34, *p* < 0.001), indicating that the DSS model of ulcerative colitis in mice was successfully established and that intestinal inflammation had not recovered even after stopping DSS for seven days with normal circadian rhythm and sufficient sleep time.

On the other hand, sleep interruption increased intestinal inflammation and delayed intestinal repair. Compared with normal sleep (DSS group), the mice treated with 7 days of fragmented sleep (DSS + SF group) had apparent colon inflammation, including significant weight loss (−3.53 ± 0.54 vs. −1.96 ± 0.34 gm, *p* = 0.045), increased DAI (5.25 ± 0.41 vs. 3.25 ± 0.37, *p* = 0.015), shortened colon length (5.81 ± 0.13 vs. 6.27 ± 0.15 cm, *p* = 0.026), and increased histological score (18.09 ± 1.01 vs. 13.27 ± 1.69, *p* = 0.040). One mouse had to be sacrificed early on the sixth day of sleep deprivation due to severe underweight. These findings indicate that lack of sleep affects the recovery process of the inflamed intestines.

Furthermore, we used EA to stimulate another group treated with DSS and sleep fragmentation (DSS + SF + EA). After seven days of acupoint stimulation for 15 min (12 Hz and 1 degree of intensity (pulse voltage 1.5 Vp-p)), there was significant recovery from the body weight decline (−1.64 ± 0.35 vs. −3.53 ± 0.54 gm, *p* = 0.021), a lower DAI score (2.50 ± 0.46 vs. 5.25 ± 0.41, *p* < 0.001), a smaller decline in the colon length (6.58 ± 0.19 vs. 5.81 ± 0.13 cm, *p* = 0.003), and a significant recovery in the histologic inflammation score (10.45 ± 1.54 vs. 18.09 ± 1.01, *p* = 0.001). Based on these data, we know that EA has protective effects in mice with DSS-induced ulcerative colitis and sleep fragmentation.

Given that sleep fragmentation was an additional factor, we designed an additional group (the SF group) to confirm whether sleep fragmentation itself affects inflammatory responses. We found no significant difference from the control group regarding body weight (0.65 ± 0.24 vs. 0.84 ± 0.14 gm, *p* = 0.755), DAI (0.00 ± 0.00 vs. 0.00 ± 0.00, *p* = 1.000) and colon length shortening (7.40 ± 0.09 vs. 7.20 ± 0.18 cm, *p* = 0.282), except that the SF group had a higher histologic inflammation score than the control group (4.00 ± 0.58 vs. 1.64 ± 0.34, *p* = 0.005). Therefore, although sleep fragmentation itself caused mild mucosal proliferation and inflammatory cell infiltration, it did not play a major role in causing intestinal inflammation. However, if inflammation of the intestine was already present, sleep fragmentation did undermine recovery.

In addition, we found that there were not only more infiltrating inflammatory cells but also fewer goblet cells under H&E staining in the DSS group (Figure 1E). The histologic change was even more severe in the DSS + SF group. However, in the mice that received EA, the recovery from inflammation was more obvious, the goblet cells were maintained for a longer period and did not disappear, and the tissue structure remained more stable. In addition, there was no obvious difference between the control group and the SF group except for slight mucosal proliferation and inflammatory infiltration in the SF group.

**Figure 2:**
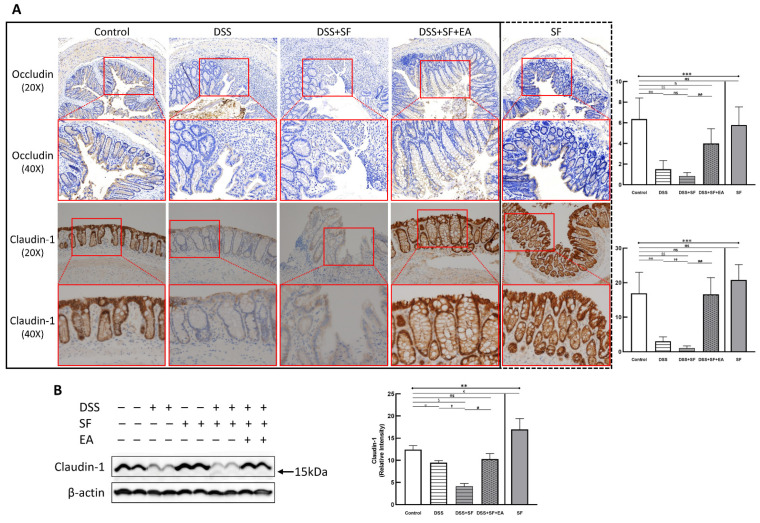
Tight junction proteins in mice colon during DSS colitis with sleep fragmentation. (**A**) Immunohistochemical (IHC) staining of occludin and claudin-1 in colon sections. Upper part: expression of occludin in colon tissue of different groups at 200× & 400× magnification. Comparison of the occludin in five groups was shown in the right upper figure. Lower part: expression of claudin-1 in colon tissue of different groups at 200× and 400× magnification. Comparison of the claudin-1 in five groups was shown in the right lower figure. Quantification of area percentage of IHC staining by true color image analysis with the application of adjusted thresholds. (**B**) Western blot analysis of claudin-1 and β-actin (loading control) in colon homogenates. Right graph indicates quantification relative to β-actin. The uncropped western blot figures were presented in Figure S1. The densitometry readings/intensity ratio of claudin-1 in western blots in DSS-colitis mice with sleep fragmentation were presented in Table S3. * Stands for a result of five groups comparison (represented by bold line segments with endpoints). ✠, ✟, #, $, &, and € represent the results of control group versus DSS group, DSS group versus DSS + SF group, DSS + SF group versus DSS + SF + EA group, control group versus DSS + SF group, control group versus DSS + SF + EA group, and control group versus SF group, respectively (represented as a thin line without endpoints). *, ✠, ✟, #, $, &, €, *p* < 0.05; ✠✠, ✟✟, ##, $$, *p* < 0.01; ***, *p* < 0.001; ns, no significance. Data were presented as mean ± SEM of repeated adjusted thresholds in each group. DSS, dextran sodium sulfate; SF, sleep fragmentation; EA, electroacupuncture.

Immunohistochemistry (IHC) staining of the tight junction (TJ) protein claudin-1 (lower part of Figure 2A) and occludin (upper part of Figure 2A) found apparent TJ protein expressions in the intestinal mucosa of normal mice to maintain the integrity of the intestinal epithelium. In the DSS group, the expressions of claudin-1 and occludin disappeared (both *p* = 0.002 compared to the control group), even when the intestinal epithelial structure had not been chemically destroyed under DSS management. Furthermore, the appearance of claudin-1 was even more significantly reduced under DSS combined with sleep fragmentation (DSS + SF vs. DSS, *p* = 0.004). In contrast, the performance of claudin-1 and occludin were restored considerably after EA intervention (DSS + SF + EA vs. DSS + SF, both *p* = 0.002). At the same time, simple sleep fragmentation (SF group) did not affect claudin-1 and occludin (*p* = 0.240 and *p* = 0.818 compared to the control group, respectively). Similar trends of claudin-1 can be confirmed through western blot (Figure 2B), which significantly decreased after giving DSS and adding SF, and recovered after EA management. Besides, SF presented an obvious upregulation.

### 3.2. Sleep Fragmentation Disturbs Immunology in Colonic Layers in the Normal State Rather than the Inflammatory Condition

We assessed the response of “sleep fragmentation” to the body in the normal and inflamed states through the multiplex immunological test of colonic tissue (Table 1). Not surprisingly, DSS is a chemical inducer of epithelial damage, leading to intestinal inflammation, resulting in a significant increase in proinflammatory cytokines compared to normal (*p* = 0.021 in IL-1β and IL-17A). Under this condition, fragmented sleep only further significantly increased the proinflammatory cytokine IL-1β in inflammatory mice compared with the DSS group (*p* = 0.021), but the anti-inflammatory cytokine remained unchanged. However, both pro- and anti-inflammatory cytokines were significantly elevated in normal mice after sleep fragmentation (*p* = 0.021 for all markers). Detailed colonic immunoassay results in five groups were shown in Table S4.

### 3.3. EA Modulated Inflammatory and Immune Reaction in DSS-Mice with Sleep Fragmentation

**Figure 3:**
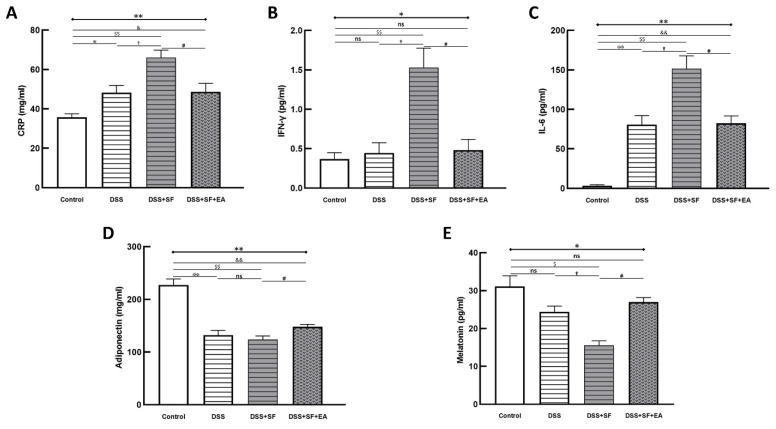
Electroacupuncture decreases plasma inflammatory marker/cytokines and increases adiponectin/melatonin in dextran sulfate sodium (DSS)-induced colitis with sleep fragmented C57BL/6 mice. (**A**) C-reactive protein (CRP), as an inflammatory biomarker, was elevated in DSS group and aggravated in DSS + SF group, then declined in DSS + SF + EA group. Similar trends were observed in other proinflammatory cytokines, including IFN-γ (**B**) and IL-6 (**C**). Adiponectin (**D**) and melatonin (**E**) level were decreased in DSS/DSS + SF groups and increased in DSS + SF + EA group. * Stands for a result of four groups comparison (represented by bold line segments with endpoints). ✠, ✟, #, $ and & represent the results of control group versus DSS group, DSS group versus DSS + SF group, DSS + SF group versus DSS + SF + EA group, control group versus DSS + SF group, and control group versus DSS + SF + EA group, respectively (represented as a thin line without endpoints). *, ✠, ✟, #, $, &, *p* < 0.05; **, ✠✠, $$, &&, *p* < 0.01; ns, no significance. Data were presented as mean ± SEM of five mice in each group. DSS, dextran sodium sulfate; SF, sleep fragmentation; EA, electroacupuncture.

We compared the acute inflammatory protein CRP, which was elevated in the DSS group and even more so in the DSS + SF group (DSS vs. control: *p* = 0.032; DSS + SF vs. DSS: *p* = 0.016), and it was significantly decreased (DSS + SF + EA vs. DSS + SF: *p* = 0.032, as shown in Figure 3A) in the EA group (DSS + SF + EA).

Regarding related proinflammatory cytokines in plasma, in the DSS group, after a week of regular sleep and water without DSS, the level of IFN-γ only slightly increased compared to that in the control group (*p* = 1.000), while the level of IL-6 still increased significantly (*p* = 0.008). However, both the IFN-γ and IL-6 levels in the DSS + SF group were significantly elevated compared with those in the control group (*p* = 0.008) and the DSS group (*p* = 0.016) and were significantly decreased in the EA group (DSS + SF + EA vs. DSS + SF, both *p* = 0.016, see Figure 3B,C). Nevertheless, when comparing the EA group to the control group, there was no significant difference in the level of IFN-γ (*p* = 0.841), but that of IL-6 was still high (*p* = 0.008).

In contrast, the level of adiponectin decreased significantly in the DSS group and DSS + SF group (*p* = 0.008, see Figure 3D). Acupuncture in the DSS + SF + EA group reversed the decrease in the level of adiponectin, which was significantly different from that in the DSS + SF group (*p* = 0.016).

Because melatonin has been reported to eliminate inflammatory activity in IBD, and sleep fragmentation itself has a strong association with melatonin secretion, we quantified melatonin in the plasma of each group. We found that with DSS, the level of melatonin in plasma tended to decline (*p* = 0.200), which reached significance in the DSS + SF group (compared to the control and DSS groups, both *p* = 0.029). However, the decrease in the melatonin level in the DSS + SF + EA group was reversed significantly compared to that in the DSS + SF group (*p* = 0.029, see Figure 3E) and did not differ from that in the control group (*p* = 0.343).

**Figure 4:**
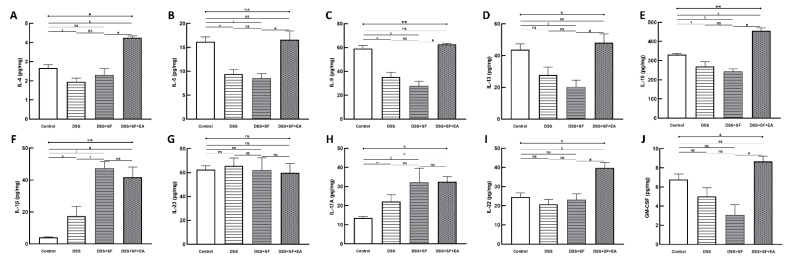
Electroacupuncture effect in colonic immunoassay in dextran sulfate sodium (DSS)-induced colitis in sleep fragmented C57BL/6 mice. (**A**) IL-4, (**B**) IL-5, (**C**) IL-9, (**D**) IL-13, (**E**) IL-10, (**F**) IL-1β, (**G**) IL-23, (**H**) IL-17A, (**I**) IL-22, and (**J**) GS-CSF concentration in colon tissue in four groups. Electroacupuncture performed significant increasement in Th2/ILC2 related cytokines (**A**–**E**), as well as ILC3-derived IL-22 and GM-CSF (**I**,**J**), but showed no obvious change over IL-1β, IL-23, and IL-17A (**F**–**H**). * Stands for a result of four groups comparison (represented by bold line segments with endpoints). ✠, ✟, #, $ and & represent the results of control group versus DSS group, DSS group versus DSS + SF group, DSS + SF group versus DSS + SF + EA group, control group versus DSS + SF group, and control group versus DSS + SF + EA group, respectively (represented as a thin line without endpoints). *, ✠, ✟, #, $, &, *p* < 0.05; **, *p* < 0.01; ns, no significance. Data were presented as mean ± SEM of four mice in each group. DSS, dextran sodium sulfate; SF, sleep fragmentation; EA, electroacupuncture; IL, interleukin; GM-CSF, Granulocyte-macrophage colony-stimulating factor; ILC, innate lymphoid cells.

Not only could the inflammatory reaction modulate by EA, but also the colonic immune reaction. Multiple immunoassays of intestinal tissue were used to explore the role of immune-related cytokines in EA therapy (Figure 4). First, we found no difference between the DSS + SF + EA and DSS + SF groups in IFN-γ and TNF-α levels (shown in Table S5); these groups exhibited Th1/ILC1-derived cytokines.

On the other hand, while IL-1β, IL-23 (Figure 4F,G), and IL-17A (Figure 4H) did not differ between DSS + SF + EA and DSS + SF groups (termed ILC3-stimulating and secreting cytokines, respectively), ILC3-derived cytokines such as IL-22 (Figure 4I) and GM-CSF (Figure 4J) both were significantly increased in the DSS + SF + EA group compared with the DSS + SF group (both *p*-value were 0.021).

Interestingly, acupuncture also affected group 2 innate lymphoid cells (ILC2)-derived cytokines, and the changes were significant and consistent. Figure 4A–D shows that IL-4, IL-5, IL-9, and IL-13 in the EA group (DSS + SF + EA) were significantly recovered. Compared with the DSS + SF group, the Th2/ILC2-related cytokines mentioned above showed statistically significant differences (each *p*-value was 0.021). IL-10, a potent anti-inflammatory cytokine that exerts its function on ILC2s, was also increased significantly in the EA group (*p* = 0.021 compared to the DSS + SF group, see Figure 4E). Detailed between-group comparisons of results from the multiplex immunoassay in colonic tissue were shown in Table S5.

### 3.4. Dysbiosis Was Improved with EA Intervention in Colitis Mice with Sleep Fragmentation

Four intestinal flora samples in each group were processed for 16S metagenomics analysis. When obtaining quality filtered and nonchimeric data, we used the six software packages mentioned below for analysis and assigned each operational taxonomic unit (OTU) to a possible bacterial taxonomy category by comparing SILVA 132 ribosomal RNA (rRNA) databases. In a total of 20 stool samples, each sample was standardized to equal sequencing depths and clusters. After filtering low abundance (<0.1%) OTUs, 796 OTUs were obtained with sequence identity ≥97%.

**Figure 5:**
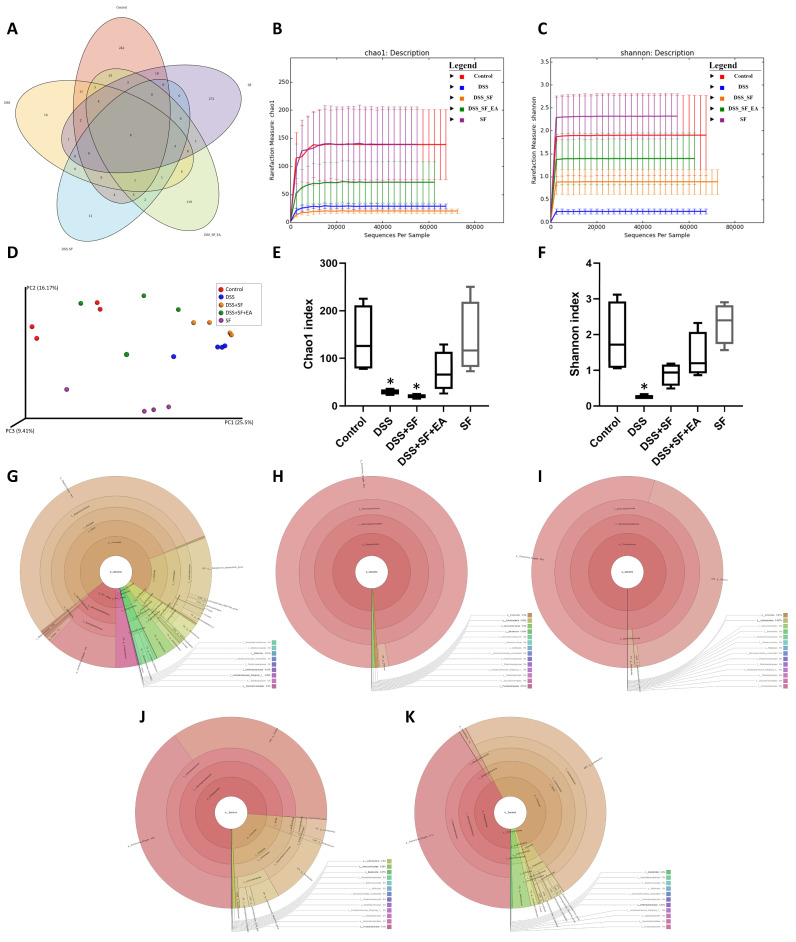
Evaluation of illumina MiSeq sequencing data showing that EA could maintain the species abundance and modulate the overall structure of gut microbiota in DSS-induced colitis with sleep fragmentation. (**A**) Venn diagram of OTUs in the five groups. (**B**) Rarefaction curves of Chao1, a measure of species richness. (**C**) Rarefaction curves of Shannon, a measure of species evenness. Rarefaction curves of OTUs sampling depth. Each curve represents a group. The sequences number is on the *X*-axis, and Chao1 and Shannon are shown on the *Y*-axis. (**D**) Principal Coordinates Analysis (PCoA) score. Each point represents a sample, plotted by a principal component on the *X*-axis and another principal component on the Y- axis, which was colored by group. (**E**) Comparison of Chao1 index in the five groups. (**F**) Comparison of Shannon index in the five groups. (**G**–**K**) In the interactive html result, circles show different classification levels from the phylum to the genus (inside to outside) in the community. Samples of the control group, DSS group, DSS + SF group, DSS + SF + EA group, and SF group were represented either individually (colored by each sample name) or grouped (colored by each group name). * Represents the results of compared with the control group. ((**A**–**K**), *n* = 4 in each group). DSS, dextran sodium sulfate; SF, sleep fragmentation; EA, electroacupuncture.

We found that the number of OTUs (Figure S2A,B) in the intestines and the alpha diversity (Figure 5B,C) of microorganisms decreased significantly with DSS administration, especially DSS combined with sleep interruption (DSS + SF), which had the lowest performance based on the metagenomics analysis among all the groups. EA treatment (DSS + SF + EA) restored the disordered flora distribution. In contrast, the species richness and species evenness were maintained in the sleep interruption (SF) group (Figure 5).

The Venn diagram in Figure 5A below shows that there were indeed very significant differences in the number of OTUs between the five groups (*p* = 0.005), and only 9 OTUs overlapped between the five groups. Even the SF and control groups had only 39 identical OTUs. However, there were still 90 OTUs consistent between the EA group (DSS + SF + EA) and the control group. In terms of the number of species, the DSS + SF group had the lowest, followed by the DSS group. The groups with the most abundant species were the control and SF groups. EA maintained the number of species reduced by the influence of DSS and sleep fragmentation.

Furthermore, the Chao1 (Figure 5B,E) and Shannon (Figure 5C,F) indexes were used to represent the richness and evenness indicators of the species, and both indexes were significantly reduced (*p* = 0.029) in the DSS group compared with the control group. Compared with the control group, the DSS + SF group had a significant decrease in richness (*p* = 0.029), but the decline in evenness did not reach a statistically significant difference (*p* = 0.200). Regardless of the EA group or the SF group, there was no significant difference in Chao1 and Shannon index from the control group. Then, we compared the effects of sleep fragmentation in normal and inflamed mice. Species richness and evenness were stable when normal mice faced sleep disturbance, but dysbiosis emerged when inflamed mice experienced sleep fragmentation (species richness decreased, but evenness increased, *p* = 0.057 and 0.029, respectively).

In the comparative analysis of sample grouping (beta diversity), Figure S2C showed a heatmap drawn by unweighted UniFrac distance, and Figure S2D presented an unweighted pair group method with arithmetic mean (UPGMA) clustering tree. We found that the distance on the heatmap between the EA group (DSS + SF + EA) and the control group (control) was relatively close, followed by the SF and the DSS + SF groups. The farthest was the DSS group. Similar results were also presented on the UPGMA cluster tree analysis, again showing that the classification of the EA group (DSS + SF + EA) and the control group were closer, followed by the SF and DSS groups. This time the most distant classification was the DSS + SF group. According to principal coordinates analysis (PCoA, see Figure 5D), principal component analysis (PCA, see Figure S2E) and partial least squares discriminant analysis (PLS-DA, see Figure S2F) between each group, the gut flora of the DSS group and the DSS + SF group deviated from the flora cluster of the control group. In contrast, the flora cluster of the EA group gradually returned to the cluster of the control group. Notably, the flora of the sleep interruption group had a trend of its own. Similar trends could be seen in the hierarchical relationship of all the gut microbiota (Figure 5G–K).

**Figure 6:**
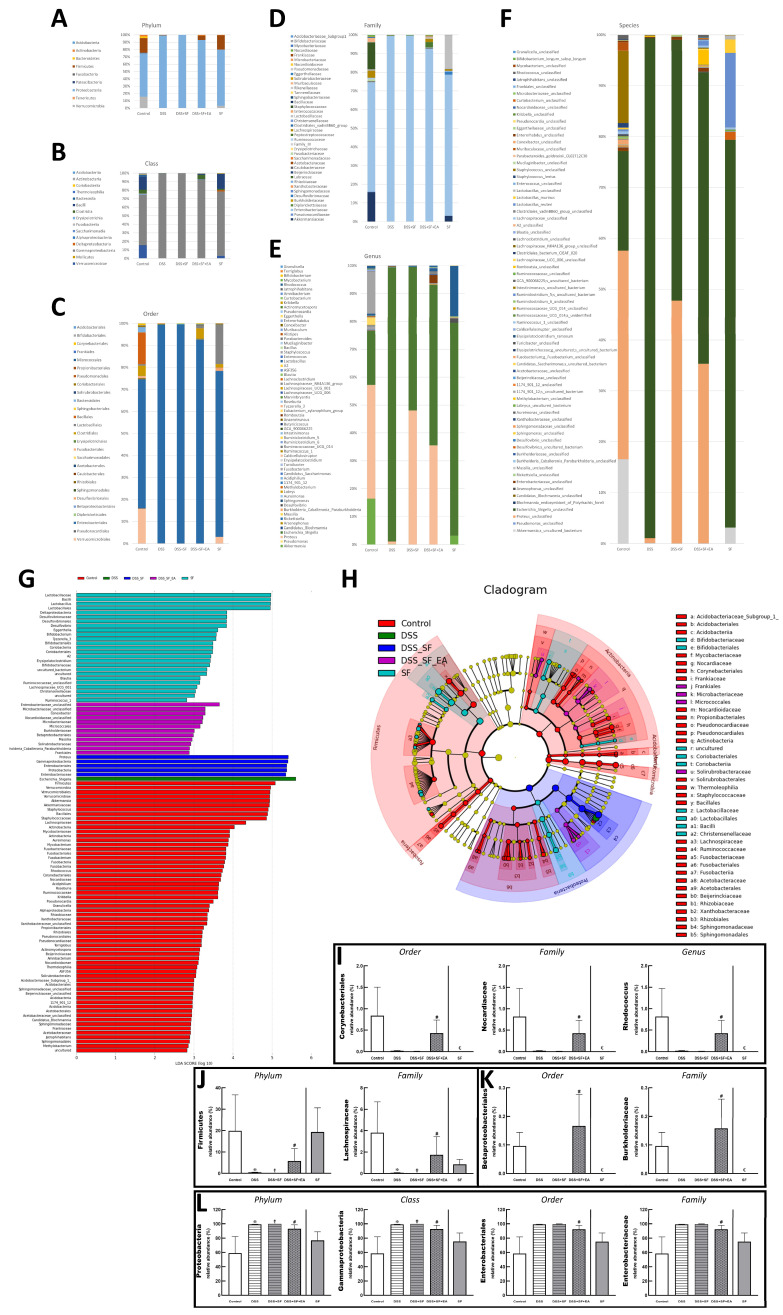
EA alters the intestinal microbial composition in DSS colitis mice with sleep fragmentation. Microbial community bar plot by (**A**) phylum, (**B**) class, (**C**) order, (**D**) family, and (**E**) genus. (**G**) LEfSe (Linear discriminant analysis effect size). Comparisons of the relative abundance at the level of bacterial phylum. Each bar represents the effect size (LDA) for a particular taxa in a certain group. The length of the bar represents a log10 transformed LDA score. The colors represent which group that taxa was found to be more abundant compared to the other group. (**H**) LEfSe Cladogram. The green-group positive-enriched taxa are indicated with a positive LDA score (green) and red-group control-enriched taxa have a negative score (red). Only the taxa with meeting a significant LDA threshold value of >2 are shown. (**I**–**L**) Comparison of five groups in the specific microorganisms and their subordinates. (**I**) Comparison of five groups in the genus *Rhodococcus* and its superior (family and order) in bar charts. (**J**) Comparison of five groups in the family *Lachnospiraceae* and its superior (family and phylum) in bar charts. (**K**) Comparison of five groups in the family *Burkholderiaceae* and its superior (family and order) in bar charts. (**L**) Comparison of five groups in the family *Enterobacteriaceae* and its superior (order, class, and phylum) in bar charts. Samples of the control group, DSS group, DSS + SF group, DSS + SF + EA group, and SF group were represented by groups (colored by each group name). ✠,✟, #, and € represent the results of control group versus DSS group, DSS group versus DSS + SF group, DSS + SF group versus DSS + SF + EA group, and control group versus SF group, respectively. ✠, ✟, #, €, *p* < 0.05. ((**A**–**L**), *n* = 4 in each group). DSS, dextran sodium sulfate; SF, sleep fragmentation; EA, electroacupuncture.

Bar charts illustrating the gut microbiota community structure, revealing the microbial species and their relative abundances. As shown in Figure 6A, the most abundant phyla in the control group were *Proteobacteria* and *Firmicutes*, and the dominant phylum became *Proteobacteria* only in both DSS and DSS + SF groups. In the EA group, *Proteobacteria* reduced, and *Firmicutes* increased, while the SF group was no different from the control group. These two phyla and *Verrucomicrobia*, *Acidobacteria*, *Actinobacteria*, and *Fusobacteria* were all meet the significant differences in the relative abundance of gut microbiota between five groups at the phylum level, but only *Proteobacteria* and *Firmicutes* had significant differences between DSS + SF and DSS + SF + EA group (*p*-value were 0.007 and 0.025, respectively, see Figure 6J,L). At the class level displayed in Figure 6B, *Gammaproteobacteria*, *Verrucomicrobiae*, *Bacilli*, *Actinobacteria*, *Fusobacteriia*, *Coriobacteriia*, *Alphaproteobacteria*, *Acidobacteriia*, *Thermoleophilia*, and *Deltaproteobacteria* (ranked by abundance in the control group) were significantly different among the five groups, and only *Gammaproteobacteria* had significant difference between DSS + SF and DSS + SF + EA group (*p* = 0.007, Figure 6L).

At the order level illustrated in Figure 6C, *Enterobacteriales* were dominant in the control group but explosively dominant in the DSS and DSS + SF groups, then rebalanced percentage in the DSS + SF + EA group (*p* = 0.043 compared to the DSS + SF group) and constant in the SF group (Figure 6L). On the flip side, *Corynebacteriales* and *Betaproteobacteriales* were tiny proportions in the control group. Still, they became scant in DSS and DSS + SF groups and obviously restored percentage in the DSS + SF + EA group (*p* = 0.043 & *p* = 0.047, respectively, compared to the DSS + SF group). Unlike usual, the ratio of these two orders in the SF group were not maintained but significantly decreased (both *p* = 0.014 when compared to the control group, see Figure 6I,K).

At the family level demonstrated in Figure 6D, continuing the trend in order level, *Enterobacteriaceae* accounted for the bulk in the control group, exploded in the DSS and DSS + SF groups, and then re-declined the percentages in the DSS + SF + EA group (*p* = 0.043 when versus DSS + SF group, Figure 6L). On the other hand, few *Nocardiaceae* and *Burkholderiaceae* were in the control group, while downward in the DSS and DSS + SF groups and upward in the DSS + SF + EA group (*p* = 0.043 and 0.047 compared with the control group, respectively. Figure 6I,K). Again, these two families in the SF group have the same trend of dramatically downhill (both *p* = 0.014 when compared to the control group, Figure 6I,K). As for *Lachnospiraceae*, a subordinate of *Firmicutes* and ranked fourth in abundance in the control group (after *Enterobacteriaceae*, *Akkermansiaceae*, and *Staphylococcaceae*), its proportion also decreased significantly in the DSS and DSS + SF groups and rebounded significantly after EA (DSS + SF + EA group, *p* = 0.047, see Figure 6J). The ratio in the SF group was between the control and DSS + SF + EA groups.

While the genus level (Figure 6E) excludes some genera that changed significantly but are unclassified (e.g., *unclassified Enterobacteriaceae* counted to 0.61%, others were all lower than 0.08% in the control group), still more than thirty genera had significant difference between five groups. *Rhodococcus* was found at significantly lower levels in the DSS and DSS + SF groups compared with that in the control and DSS + SF + EA group (*p* = 0.043). This genus continued the trend of its superior in the family and order, significantly becoming a scant proportion in the SF group (compared to the control group, *p*-value was 0.043, Figure 6I).

During gut flora analysis at the species level (Figure 6F), some specific bacteria identified had significant differences between five groups, including *Bifidobacterium longum subsp longum*, *Lactobacillus murinus*, *Lactobacillus reuteri*, *Clostridiales bacterium CIEAF 020*, *Erysipelatoclostridium ramosum*. However, no specific species have been recognized with a significant difference between DSS + SF and DSS +SF + EA, except for some unclassified species such as *Rhodococcus* sp.

Finally, we used linear discriminant analysis (LDA) to compare the microbial species between the groups. The results were shown in the linear discriminant analysis effect size (LEfSe) and cladogram (Figure 6G,H). When accounting for the majority of the microorganisms, the control group had the most numerous species, dominated by *Firmicutes*, followed by *Verrucomicrobia*, including *Akkermansiaceae* and *Akkermansia*. The SF group had the second greatest number of species and showed more *Deltaproteobacteria*, but the most numerous microorganisms were still *Lactobacillaceae* and its subordinate *Lactobacillus*, as well as *Bacillus*. The following group was the EA group (DSS + SF + EA), which ranked third among the five groups and exhibited far more species than the DSS and DSS + SF groups. However, many microorganisms were still unclassified bacterial species, such as *unclassified Enterobacteriaceae*, *unclassified Microbacteriaceae*, and *unclassified Nocardioidaceae*. Others were found in very small numbers, such as *Conexibacter* (originally only accounted for 0.010%), *Microbacteriaceae* (0.08%), *Micrococcales* (0.083%), *Burkholderiaceae* (0.053%), *Betaproteobacteriales* (0.055%), *Massilia* (0.016%), *Solirubrobacteraceae* (0.011%), *holderia Caballeronia Paraburkholderia* (0.029%), and *Frankiales* (0.051%). Ranked fourth was the DSS + SF group. In this group, *Proteus*, *Gammaproteobacteria*, *Enterobacteriales*, *Proteobacteria*, and *Enterobacteriaceae* were the dominant bacteria. The least difference in the number of bacteria was observed for the DSS group, and only *Escherichia Shigella* was the main species.

### 3.5. Vasoactive Intestinal Peptide (VIP) Performance on Intestinal Homeostasis with EA Treatment

EA is purely physical stimulation. It neither regulates prebiotics by administering or changing dietary metabolites nor affects the intestinal flora by directly supplementing probiotics. EA can improve intestinal inflammation and even reconstruct the abundance of gut microbiota. It is speculated that the possible mechanism is through the enteric nervous system (ENS), which affects the intestinal microenvironment and mucosal immune system, and the ILCs may act as a critical link between the nervous system and microbiota in intestinal networks. According to the immunoassay in colonic tissue, the ILC2 and partial ILC3-derived cytokines were elevated in mice after EA treatment. We selected VIP, a nonadrenergic noncholinergic neurotransmitter that modulates ILC through the ENS, to determine its role.

**Figure 7:**
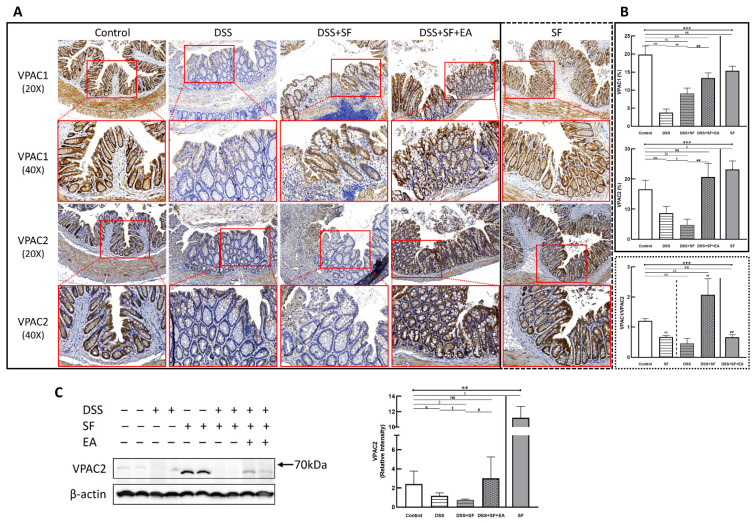
Vasoactive intestinal peptide receptors in mice colon during DSS colitis with sleep fragmentation. (**A**) Immunohistochemical (IHC) staining of VPAC1 and VPAC2 in colon sections. Upper part: expression of VPAC1 in colon tissue of different groups at 200× & 400× magnification. Comparison of the VPAC1 in five groups was shown in (**B**) right upper panel. (**A**) Lower part: expression of VPAC2 in colon tissue of different groups at 200× & 400× magnification. Comparison of the VPAC2 in five groups was shown in (**B**) right middle panel. Comparison of VPAC1/VPAC2 ratios by sleep fragmentation in normal and inflamed state was shown in (**B**) right lower panel. Quantification of area percentage of IHC staining by true color image analysis with the application of adjusted thresholds. (**C**) Western blot analysis of VPAC2 and β-actin (loading control) in colon homogenates. Right graph indicates quantification relative to β-actin. * Stands for a result of five groups comparison (represented by bold line segments with endpoints) The uncropped western blot figures were presented in Figure S1. The densitometry readings/intensity ratio of VPAC2 in western blots in DSS-colitis mice with sleep fragmentation were presented in Table S3. ✠, ✟, #, $, & and € represent the results of control group versus DSS group, DSS group versus DSS + SF group, DSS + SF group versus DSS + SF + EA group, control group versus DSS + SF group, control group versus DSS + SF + EA group, and control group versus SF group, respectively (represented as a thin line without endpoints).*, ✠, ✟, #, $, &, €, *p* < 0.05; **, ✠✠, ✟✟, ##, $$, &&, €€ *p* < 0.01; ***, *p* < 0.001; ns, no significance. Data were presented as mean ± SEM of repeated adjusted thresholds in each group. VPAC1, vasoactive intestinal peptide (VIP) type 1 receptor; VPAC2, vasoactive intestinal peptide (VIP) type 2 receptor; DSS, dextran sodium sulfate; SF, sleep fragmentation; EA, electroacupuncture.

VIP type 2 receptor (VPAC2) shows membrane expression in most tissues, including intestinal endothelial cells and smooth muscle cells, and VIP type 1 receptor (VPAC1) also shows protein expression along the length of the mouse intestine. Through IHC staining of VPAC2 (lower part of Figure 7A), we found a similar trend to TJ proteins, especially claudin-1. That is, the intestinal endothelial cells of normal mice had a prominent VPAC2 presentation. In contrast, the staining of VPAC2 in DSS (DSS group) and DSS combined with sleep fragmentation (DSS + SF group) decreased gradually (both *p* = 0.004, Figure 7B middle). After the intervention with EA, it recovered and even tended to be overexpressed (*p* = 0.004 compared to the DSS + SF group, and *p* = 0.109 compared to the control group, Figure 7B middle). Fragmented sleep did not lead to a decrease in VPAC2 receptors but also had a higher level than the EA group, reaching a significant overexpression (*p* = 0.010 compared to the control group, Figure 7B middle). Similar trends of VPAC2 could be confirmed through western blot (Figure 7C), which significantly decreased after giving DSS and adding SF, and recovered after EA management. SF presented a great upregulation.

On the other hand, the VPAC1 expression (upper part of Figure 7A) in the normal mice was still prominent, and its level in the DSS group became the lowest (*p* = 0.004, Figure 7B upper), then upregulated in the DSS + SF group (both *p* = 0.004 compared to the DSS group and the control group, Figure 7B upper), and became more increased after EA treatment (both *p* = 0.004 compared to the DSS group and the control group, Figure 7B upper). This time, both DSS + SF + EA and SF groups did not show overexpression compared to the control group but were still significantly lower than the control group (*p* = 0.004 & *p* = 0.006, respectively, Figure 7B upper). Notably, sleep fragmentation appears to have opposed manifestations in VPAC1 and VPAC2 when faced with normal and inflamed conditions. Sleep fragmentation significantly downregulated VPAC2 in colitis but upregulated it in normal mice (Figure 7B middle). Conversely, sleep fragmentation significantly upregulated VPAC1 in colitis but downregulated it in normal mice (Figure 7B upper). This finding could be assessed with the VPAC1/VPAC2 ratio shown in the lower panel of Figure 7B.

## 4. Discussion

EA treatment can effectively reduce intestinal inflammation and affect the gut microbiota in ulcerative colitis with sleep fragmentation. The average DAI of the mice, the macroscopic colon length, and the microscopic histopathology were significantly improved in the EA group. At the same time, EA recovered the aggravated dysbiosis, preserved tight junction proteins, and upregulated the expression of VIP receptors.

DSS-induced ulcerative colitis in mice is the pathological core and a vital evaluation aspect of the entire experiment. At the same time, sleep interruption has been confirmed to affect the body’s recovery, including the production of inflammation [38]. Similar experiments have also verified in animals that light sleep deprivation can cause intestinal barrier malfunction, affecting melatonin and intestinal flora disorders [39]. Short-term or long-term sleep interruptions affect the gut microbiota of rats and even change the morphology of the intestine [40]. In our results, simple sleep fragmentation only changed intestinal cytokines but did not affect intestinal morphology or defecation function. However, our findings confirm that sleep deprivation will aggravate rodent ulcerative colitis [29,31] and even increase mortality [41]. Sleep plays an important role in IBD because sleep deprivation itself is quite a considerable stress, and experimental methods that interfere with sleep (such as horizontal movement of a crossbar) may also cause stress on animals. Stress can exacerbate inflammation in the digestive tract [31]. In addition, sleep also affects the circadian rhythm and inflammatory cytokines through immunity [42]. Both affect intestinal microbes.

Intestinal epithelial cells (IECs) formed various kinds of mucosal layers separating gut microbes and immune cells to prevent the following overreaction of inflammation, which can be viewed as a critical barrier to intestinal homeostasis. It has been proven that dysbiosis is associated with intestinal inflammation in animal models of colitis [43]. Still, studies have also found that both declines of tight junction proteins (like ZO-1, claudin-1, occludin) and increasing permeability (leaky gut) appear forward to severe gut inflammation. In the model of DSS-induced colitis, the alteration of tight junction is not a secondary result of inflammation but might facilitate the inflammation formation of colitis [44]. Tight junctions and adherens junctions compose the apical junctional complex on the top of the intestinal epithelium. Thus, it can control the epithelial permeability and further keep intestinal homeostasis. In this process, IECs expressed various recognition receptors lie toll-like receptors (TLRs) to sense the composition of bacteria and further drive the production of antimicrobial molecules with TLR4/MyD88 signal and NOD2 signal [45]. Our previous studies found that acupuncture can improve intestinal inflammation through the signal transmission of TLR4/MyD88, while maintaining the tight junction of the intestine [46]. The above experimental results have proved that sleep disruption would deteriorate DSS colitis’s severity, followed by TJ damage (eg. claudin-1) and increased proinflammatory mediators (including CRP, IFN-γ, and IL-6). Repeated electroacupuncture could improve DSS-induced colitis, repair TJ damage (both claudin-1 and occludin) and inhibit proinflammatory mediators.

Research on the gut microbiota has surged in the past two decades. As early as 2004, it was reported that mucosal inflammation in IBD was associated with the loss of normal anaerobic bacteria [47]. In recent years, abnormal microbiota composition and decreased complexity of the gut microbial ecosystem have usually been observed in patients with Cohn’s disease and ulcerative colitis [48]. However, whether changes in intestinal microbes are the cause or effect of IBD remains to be elucidated [49]. In animal models, DSS-induced colitis has been shown to cause intestinal flora disorders in mice, including reducing bacterial species richness and changing bacterial community composition [50]. Animal experiments have shown that EA can alleviate the symptoms and signs of ulcerative colitis and has a partial recovery effect on the intestinal flora [51]. For example, the content of *Clostridium bifermentans* decreases, and *Lachnospiraceae bacterium* and *Lactobacillus* sp. increase. Even after constantly administering DSS to keeping gut inflammation, EA could maintain colon length and daily body weight. PCA, Venn diagrams, and similarity trees showed the effect of EA on modifying the components and abundance of intestinal flora. The aforementioned effects of EA on the gut microbiota can also be found in our data. Not only that, we found EA could modulate the abundance of *Enterobacteriaceae*, which has been identified as its role in IBD pathogenesis and progression [52].

Increasing evidence has shown that intestinal microbiota is also associated with sleep quality. A recent sleep deprivation study confirmed that sleep efficiency (SE) and total sleep time (TST) were positively correlated with, and wake after sleep onset (WASO) was negatively correlated with, total microbiome diversity. In addition, there was a positive correlation between total microbiome diversity and IL-6. IL-6 has previously been noted to affect sleep [53]. IL-6 could increase SE in human studies but has a relatively minor impact on WASO in sleep interruption. This result confirmed a connection between the composition of the gut microbiome and sleep physiology, leading the author to propose the possibility of manipulating the gut microbiota to improve sleep [54]. However, our findings clearly illustrated that EA reduced the IL-6 level in plasma and simultaneously recovered the abundance and diversity of microbiomes. This result indicates that IL-6 is not so much a pleiotropic cytokine that increases sleep. It is more accurate to understand IL-6 as a compensation product that increases sleep drive, explaining why IL-6 promotes SE. Recently, study showed both SF and dark disturbance in mice produced significant alterations in the microbiota compared to home cage mice, and SF produced marked neuroinflammation with influenced immune mucosal barrier, which was not presented in the TJ protein and the gut microbiota in our SF group. Possible reasons are different protocols, as well as different degrees of SF [16].

The intestinal mucosa affects the gut microbiota [55], and intestinal epithelium integrity is tightly associated with mucosal immune function [56]. Thus, the microbiota-immune axis has become a popular topic for research [57,58]. Intestinal mucosa has a powerful ability to modulate the immune response. For example, mucosal surface defense invasion of many pathogens and other immune-stimulating factors in intestinal lumen throughout a lifetime, and even more, prevent organs from further damage to uncontrolled immune response. In other words, immunity is determined by microbiota [59]. Interaction of microorganisms and immunity began from the initial of lives. When the mucosal surface is exposed to microorganisms, immune homeostasis and defending mechanisms are established. Recently, innate lymphoid cells (ILCs), which exist mainly in mucosal tissue, have been shown to play important roles in initiating the proper immune response after pathogen infection [60]. In mice, it is known that ILCs comprise “adaptive” cytokines such as IL-22, GM-CSF, and IL-5, which are expressed at higher levels than conventional T cells during steady-state conditions [61]. ILC3s are known to be involved in complex sensory circuits to integrate microbial and dietary cues and enhance mucosal homeostasis. ILC3-derived IL-22 acts on epithelial cells to enhance intestinal barrier integrity [62,63]. In the intestinal tract, ILC2s are more homogeneous cells than ILC1s and ILC3s. Group 2 ILCs express transcription factors called GATA3 and produce type 2 cytokines such as IL-4, IL-5, IL-9, and IL-13. ILC2s not only play essential roles in the clearance of helminths [64] and viral infection [65] and the progression of allergic asthma [66,67], but ILC2 also elicits beneficial host responses to pathogens and mucosal damage [68]. ILC2 shares cytokine and transcription factor expression with CD4+ Th2 cells, but the functional diversity of the ILC2 lineage has yet to be fully explored.

Some research mentions that the formation of type 2 cytokines like IL-4, IL-5, IL-9, and IL-13 are associated with the severity of IBD [69,70]. An experimental model showed that reducing central cytokines IL-4 and IL-13 could control gut inflammation through the type 2 proinflammatory pathway of the gut mucosa. However, recent data has suggested this paradigm is not relatively so straightforward. A study showed that decreasing IL-13 does not have treating effects. Besides, ILC2s can secrete amphiregulin (AREG) against intestinal damage and inflammation in acute DSS colitis. It could explain that DSS colitis belongs to Th1 models in mucosal inflammation [71], and some studies have shown that Th2 cytokine release protects mice with DSS-induced colitis [72]. We knew that ILC2 participated in the pathogenic process of IBD [73], but the function of ILC2 in IBD has still not been fully understood.

In our experiment, the results of the multiplex immunoassay showed a partial change in ILC3-associated cytokines such as IL-22 and GM-CSF, but the more noticeable difference was in ILC2-derived cytokines, including IL-10, which could modulate DSS-induced colitis through a macrophage–ROS–NO axis [74]. The performance of ILC2 is affected by many signals. ILC2 can activate by alarmins (IL-33, IL-25, and TSLP), cytokines (IL-2, IL-4, IL-7, and IL-9), and some lipid mediators (PGD2, LTD4 and LXA4) [75]. Additionally, ILC2 can be activated by VIP secreted by the ENS, and circadian rhythms regulate its expression [76,77]. On the other hand, some studies have also pointed out that VIP secreted by the ENS also affects ILC3, which is related to the immunity of the intestinal mucosa [78,79], and diet regulates its expression and affects the intestinal barrier [80]. This finding indicates that the interaction between ILCs and neuronal cells may have a profound biological impact on tissue homeostasis [75], and the neuropeptide VIP potentiates both ILC2 and ILC3 immunity in response to circadian and feeding [81]. The VIP axis has clinical utility and has been reported in inflammatory and autoimmune diseases, including IBD [82]. Therefore, one possible reason explained the EA effect on dysbiosis was VIP mediated TJ reservation [83], which could be confirmed in our results.

VIP performance in the intestinal tract depends on the expression patterns of VIP receptors VPAC1 and VPAC2, the latter of which is closely related to ILC2, leading to its role in metabolism [84]. Although both VIP receptor subtypes are abundantly expressed in the gastrointestinal tract, the two VIP receptors show complementary expression patterns in the small intestine. Moreover, research points out that different subtypes of VIP receptors may indicate different disease development because the effects of VIP in IBD are controversial either in human research or in animal models [85]. According to current research, VPAC1 expression consists of a long-lasting baseline, and VPAC2 is only induced after activation. Therefore, a detailed analysis of VPAC1/VPAC2 levels can provide information on the dominant immune cell subsets at different stages. Regarding its function, an interesting difference was recently found in VIP knockout mice. By comparing the difference between VPAC2 knockout mice and T cell-specific transfer mice, it was shown that VPAC2 tends to lead VIP to the Th2 cell response. Hence, it has been reported that in VPAC2-deficient mice with experimental autoimmune encephalomyelitis, proinflammatory cytokines (TNF-α, IL-6, IFN-γ (Th1), and IL-17 (Th17)) increased, and anti-inflammatory cytokines (IL-10, TGF-β, and IL-4 (Th2)) decreased [86]. On the other hand, the mechanisms of VPAC1 have not been completely clarified. In experiments using macrophages and microglial cells cultured in vitro, VPAC1 is the receptor inducing the anti-inflammatory effects of VIP, but the mechanism is not entirely clear. As for the streptozotocin-induced diabetic mice [87], theVPAC1 agonist displayed a stronger anti-inflammatory effect. However, when talking about DSS colitis, VPAC1 showed upregulation in murine colitis [88], and VPAC1 and VPAC2 knockout mice appear to have the reverse appearance: clinical symptoms are milder in VPAC1 knockout mice and more severe in VPAC2 knockout mice, which could be relieved by suppression of VPAC1 signals. This means VPAC1 increases inflammatory signals in the intestine potentially, while VPAC2 signals alleviate clinical disease obviously [89]. Our results showed significantly upregulated VPAC2 expression after EA intervention, but the absolute VPAC1 expressions in five groups have no definite trend. It could be because the mice in the DSS group were under a rapid recovery status with no more DSS in the last 7 days and had sufficient sleep. Thus, we think VPAC1/VPAC2 could be seen as a breakthrough load, indicating the possible trend of colitis severity, which could be calculated from our results.

Adiponectin, secreted by mesenteric adipose tissue, has a variety of biological functions in intestinal inflammation. Recent studies have shown that it plays a key role in intestinal epithelium and inflammation. Adiponectin not only inhibits macrophage infiltration and proinflammatory cytokine release [90], but also maintains intestinal homeostasis and improves intestinal barrier integrity [91]. In some DSS mice models, adiponectin can improve the colon’s tight junction protein expression (ZO-1 and claudin-1). These three mechanisms could be confirmed in our model after electroacupuncture intervention, which was also coordinated with plasma adiponectin levels. Therefore, modulation of adiponectin may be another possible mechanism by which EA attenuates the severity of colitis in DSS mice. Another reason that sleep deprivation affects IBD is melatonin. There is a strong link between sleep deprivation and melatonin levels in an organism. Plasma melatonin was significantly reduced in sleep-deprived mice compared to normal mice, leading to intestinal barrier damage and dysbiosis. Studies indicate that it could reduce probiotics such as *Akkermansia*, *Bacteroides*, and *Faecalibacterium*, associated with significantly increasing pathogenic microorganisms such as *Aeromonas* [39]. In our results, although melatonin level declines in the DSS group, it is without a significant difference to the control group. However, melatonin level drops significantly in the sleep-fragmented group, accompanied by elevated inflammatory cytokines such as IL-1β and IL-6. EA ST36 reversed melatonin reduction while improving inflammatory responses, even with marked adiponectin elevations. These results are consistent with the reports that melatonin ameliorates sleep-fragmented ulcerative colitis in mice by increasing adiponectin [92]. In addition, one study showed that melatonin inhibits Gram(+) bacteria through TLR4 signaling to increase goblet cells, Reg3β, and the ratio of *Firmicutes* to *Bacteroidetes*. The effects of melatonin in colitis may be related to detecting bacteria in the gut lumen with TLR4, alteration of goblet cells, and regulation of antimicrobial peptides (AMPs) [93]. These results are also consistent with our study.

It is worth investigating further that while we know that sleep fragmentation does interfere with the organism, it appears to have two distinct responses in healthy bodies and inflammatory pathological states. Studies have found that 5 days of sleep restriction in healthy individuals will shift toward Th2 activity, which means a lower IL-2/IL-4 ratio [94]. On the other hand, it has also been suggested that sleep deprivation may lead to decreased neutrophil phagocytosis and NADPH oxidase activity, as well as decreased CD4+ T cell levels, which are associated with changes in Th1-related chemokine balance [95]. Our experiments found that sleep interruption in normal mice only had a slightly higher histological score than the control group, but had no effect on body weight change, colon length, disease activity index, or TJ protein expression. On the other hand, sleep interruption slightly increases the species evenness of gut microbes, making a different trend in normal and colitis mice, especially for some specific species such as *Burkholderiaceae* and *Rhodococcus*. Unlike SF in inflamed status showed only proinflammatory cytokines exacerbations, both pro- and anti-inflammatory cytokines significantly increased in normal mouse colon tissue after sleep disruption. As for the VIP receptors, sleep fragmentation seems to have opposed manifestations in VPAC1 and VPAC2 when faced with normal conditions. The VPAC1/VPAC2 ratio, implying a cut-point for intestinal inflammation breakthrough, became more protective in normal mice but more fragile in inflamed colons. This suggests that sleep interruptions may allow normal mice to be in a compensatory immune state, leading to mild mucosal hyperplasia or infiltration of intestinal inflammatory cells, but not to full-blown intestinal inflammation. Although the results appear inconsistent with most sleep studies showing that sleep deprivation reduces immune cells, such as lymphocyte proliferation [96], these differences may be related to the type and duration of sleep deprivation. It is important to note that such findings are only the result of short-term sleep deprivation and cannot be directly assumed to be the same as those of chronic sleep deprivation.

Many articles have supported the effect of sleep on inflammation and cascade immune responses. Though some said that the production of activating immune cells declined in some severe inflammatory conditions, experimental manipulation of sleep duration still proved that sleep regulates innate immunity and inflammatory markers [97]. For example, some research revealed that CRP and IL-6 levels elevated after 10 days of sleep deprivation (4 h per night) [98], and inflammatory gene transcription such as IL-1β, IL-6, and IL-17 increased after 7 days of sleep deprivation, without recovery after a complete rest of night [99]. Briefly, the effect of experimental sleep deprivation had not reached a definite conclusion. Partial reasons were the different sleep fragmentation methods (water bath vs. rotating platform vs. silent sweeper bar) [100], different lengths of sleep deprivation periods (continuous vs. intermittent), as well as different samples or cytokines were chosen. Even so, the presentation of the mice under sleep fragmentation in our experiment was remarkable, including more severe gut inflammation, destroying intestinal epithelial integrity, lowered TJ protein expression, and reduced richness of intestinal microbiota. Upregulated VPAC1 and downregulated VPAC2 were confirmed. At the same time, SF worsens colon tissue’s unbalanced immune performance, interferes with the immune regulation of intestinal mucosa, and increases proinflammatory immune factors. Collectively, the SF in inflamed mice showed an opposite trend to that in normal mice, which exacerbated intestinal inflammation. Normal mice have a higher threshold when there is no inflammatory response and are less likely to be disturbed by sleep interruptions. In comparison, inflamed mice have a lower threshold and are more susceptible to sleep interruptions that aggravate the initial inflammatory response. It can be seen that sleep fragmentation has different effects on organisms with different thresholds.

Our experiments uncovered a number of interesting findings, such as how EA can modulate the expression of gut TJ and VIP receptors associated with remodeling the gut microbiota. Due to the unique properties of purely physical stimulation of EA, the ENS is the most likely mediator, but a well-defined pathway should be elucidated by parasympathetic blockade or its mediated α7 nicotinic acetylcholine receptor agonists/antagonists. In addition, EA could affect mucosal barrier and immunity, such as epithelial integrity and ILCs-related cytokines, but we only obtained results from TJ proteins and multiplex immunoassays. A functional test of mucosal permeability [101,102] could help draw a more comprehensive picture of mucosal barrier. While ILCs can link the nervous system and microbiota in intestinal networks [73], if flow cytometry can be performed on the colonic mucosal lymphocytes in the following experiments, the role of immune cells in EA therapy can be more clearly understood. Finally, if we were to try to guide EA management in gut microbiota, increasing the number of fecal samples, combination with the metabolomics survey, or further adoption of fecal transplant experiments should be effective. Last but not least, sleep fragmentation deserves contemplating for its role in physiological and inflammatory conditions. Both DSS and SF may interfere with immunology in different body sites, although they primarily impact the two ends of the gut–brain axis, respectively. A detailed study design should be investigated to elucidate the individual and mutual influences between the two.

## 5. Conclusions

In a colitis mouse model with DSS administration followed by fragmented sleep to interrupt the repair of the intestine, the change in colon length and the daily body weight in the EA group presented a credible result. Additionally, the structure of the microbiota varied with the decreased intestinal inflammation status. Tight junction proteins on the intestinal epithelium may be the key mechanism in treating sleep-fragmented ulcerative colitis mice with EA, which influences VIP through the VPAC2 and further regulates intestinal mucosa immunity. This experiment demonstrates how physical stimulation can stabilize the intestinal epithelium and play important anti-inflammatory roles.

## Figures and Tables

**Table 1:** The results of the multiplex immunoassay in mice intestinal tissue under sleep fragmentation between normal and inflamed situation.

**Cytokines (pg/mg)**	**Control**	**SF**	**DSS**	**DSS + SF**
IFN-γ	2.71 ± 0.17	5.26 ± 0.39 ^€^	3.83 ± 0.77	3.23 ± 0.75
TNF-α	38.49 ± 1.46	66.29 ± 7.03 ^€^	44.42 ± 6.51	46.44 ± 6.92
IL-1β	4.09 ± 0.31	7.52 ± 0.61 ^€^	17.30 ± 6.14 ^✠^	47.26 ± 4.20 ^✟^
IL-6	46.19 ± 3.13	76.63 ± 4.70 ^€^	72.91 ± 15.70	104.41 ± 13.48
IL-10	331.56 ± 6.09	566.06 ± 41.86 ^€^	270.49 ± 24.03 ^✠^	243.02 ± 13.45
IL-23	62.47 ± 3.20	111.85 ± 11.10 ^€^	65.54 ± 6.73	61.88 ± 10.44
IL-17A	13.50 ± 0.72	27.63 ± 2.49 ^€^	22.12 ± 3.54 ^✠^	32.13 ± 7.39
IL-22	24.59 ± 2.09	42.29 ± 3.11 ^€^	20.76 ± 2.64	23.10 ± 3.21
GM-CSF	6.79 ± 0.56	12.27 ± 1.40 ^€^	5.02 ± 0.91	3.06 ± 1.08
IL-4	2.67 ± 0.17	5.24 ± 0.59 ^€^	1.95 ± 0.20 ^✠^	2.31 ± 0.34
IL-5	16.15 ± 1.05	28.58 ± 2.02 ^€^	9.40 ± 0.98 ^✠^	8.55 ± 0.92
IL-9	59.19 ± 2.43	105.32 ± 9.90 ^€^	35.26 ± 3.81 ^✠^	27.80 ± 3.83
IL-13	43.62 ± 3.77	83.38 ± 7.56 ^€^	27.82 ± 4.96 ^✠^	20.10 ± 4.40

^€^ represent the control group versus SF group; ^✠^ represent the control group versus DSS group; ^✟^ represent the DSS group versus DSS + SF group; ^€,^^✠^^,^^✟^, *p <* 0.05; *n* = 4 in each group.

## Data Availability

The data generated for this study are available from the corresponding author on reasonable request.

## Supplementary Materials

The following supporting information can be downloaded at: https://www.mdpi.com/article/10.3390/biology11070962/s1, Table S1: Calculation of disease activity index (DAI) score. Table S2: Criteria of histological scoring. Table S3: The densitometry readings/intensity ratio of claudin-1 and VPAC2 in western blots in DSS-colitis mice with sleep fragmentation. Table S4: Electroacupuncture effect in colonic immunoassay in dextran sulfate sodium (DSS)-induced colitis in sleep fragmented C57BL/6 mice. Table S5: Between-group comparisons of results from the multiplex immunoassay in colonic tissue in dextran sulfate sodium (DSS)-induced colitis in sleep fragmented C57BL/6 mice. Figure S1: Original western blot figures of claudin-1 and VPAC2 markers in DSS-colitis mice with sleep fragmentation. Figure S2: EA maintain the species abundance and modulate the overall structure of gut microbiota in DSS colitis mice with sleep fragmentation.
