# Supplementary material for: Alterations in Gut Microbiota and Upregulations of VPAC2 and Intestinal Tight Junctions Correlate with Anti-Inflammatory Effects of Electroacupuncture in Colitis Mice with Sleep Fragmentation
Source: Biology (Basel). 2022 Jun 25;11(7):962. doi: 10.3390/biology11070962 (PMC9311573; doi:10.3390/biology11070962)
Supplement: Supplementary file 1 [file biology-11-00962-s001.zip › biology-1752778-supplementary.pdf]

# Supplementary Materials

**Table S1.** Calculation of disease activity index (DAI) score [a]

| Score | Weight loss | Stool consistency | Rectal bleeding |
|---|---|---|---|
| 0 | No loss | Normal fecal pellet | No blood |
| 1 | 1-5% | | Trace hemoccult positive |
| 2 | 5-10% | Loose stool [b] | Hemoccult positive and visual pellet bleeding |
| 3 | 10-20% | | |
| 4 | >20% | Diarrhea [c] | Gross bleeding, blood around anus |

[a] DAI = (score of weight loss) + (score of stool consistency) + (score of rectal bleeding). [b] Loose, pasty, semisolid stools that did not stick to the anus. [c] Watery stools that adhered to the anus.

**Table S2.** Criteria of histological scoring

| Criteria | 0 | 1 | 2 | 3 | 4 |
|---|---|---|---|---|---|
| Goblet cell loss | 0 | <25% | 25-50% | 50-75% | 75-100% |
| Mucosal thickening | 0 | <25% | 25-50% | 50-75% | 75-100% |
| Inflammatory cells | 0 | <25% | 25-50% | 50-75% | 75-100% |
| Submucosal cell infiltration | 0 | <25% | 25-50% | 50-75% | 75-100% |
| Destruction architecture | 0 | <25% | 25-50% | 50-75% | 75-100% |
| Ulcers | 0 | <25% | 25-50% | 50-75% | 75-100% |

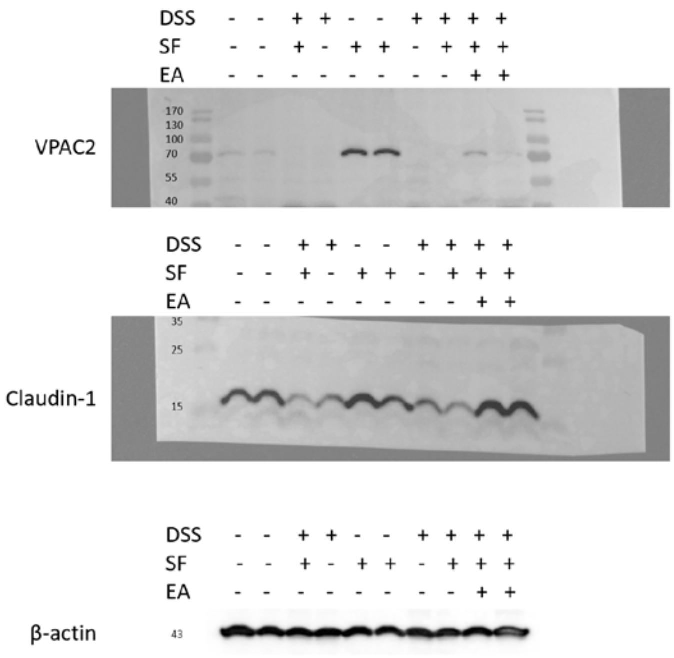


**Figure S1.** Original western blot figures of claudin-1 and VPAC2 markers in DSS-colitis mice with sleep fragmentation. C57BL/6 mice were in the presence or absence of DSS, SF, or EA administration. An equal amount of protein (40 μg) was fractionated on SDS–PAGE gels and transferred to PVDF membranes. The immunoblots showed all the bands with all molecular weight markers.

**Table S3.** The densitometry readings/intensity ratio of claudin-1 and VPAC2 in western blots in DSS-colitis mice with sleep fragmentation. The band intensity ratios were analyzed by densitometry readings/intensity ratio, using ImageJ software (NIH) and were normalized to the corresponding β-actin value. The results were expressed in relative folds change.

| Markers | Control | DSS | DSS+SF | DSS+SF+EA | SF |
|---|---|---|---|---|---|
| Claudin-1 | 12.41477 | 9.46951 | 4.15946 | 10.30464 | 16.97195 |
| VPAC2 | 2.41331 | 1.17676 | 0.75930 | 2.99923 | 11.19547 |

**Table S4.** Electroacupuncture effect in colonic immunoassay in dextran sulfate sodium (DSS)-induced colitis in sleep fragmented C57BL/6 mice.

| Cytokines (pg/mg) | Control | DSS | DSS+SF | DSS+SF+EA | SF | P-value |
|---|---|---|---|---|---|---|
| IFN-γ | 2.71±0.17 | 3.83±0.77 | 3.23±0.75 | 3.07±0.45 | 5.26±0.39€ | 0.132 |
| TNF-α | 38.49±1.46 | 44.42±6.51 | 46.44±6.92 | 38.38±4.91 | 66.29±7.03€ | 0.113 |
| IL-1β | 4.09±0.31 | 17.30±6.14✠ | 47.26±4.20$ | 41.67±6.42& | 7.52±0.61€ | 0.002** |
| IL-6 | 46.19±3.13 | 72.91±15.70 | 104.41±13.48$ | 82.88±7.71& | 76.63±4.70€ | 0.039* |
| IL-10 | 331.56±6.09 | 270.49±24.03✠ | 243.02±13.45$ | 454.95±16.58& | 566.06±41.86€ | 0.002** |
| IL-23 | 62.47±3.20 | 65.54±6.73 | 61.88±10.44 | 59.68±7.88 | 111.85±11.10€ | 0.051 |
| IL-17A | 13.50±0.72 | 22.12±3.54✠ | 32.13±7.39$ | 32.49±2.71& | 27.63±2.49€ | 0.020* |
| IL-22 | 24.59±2.09 | 20.76±2.64 | 23.10±3.21 | 39.75±2.75& | 42.29±3.11€ | 0.007** |
| GM-CSF | 6.79±0.56 | 5.02±0.91 | 3.06±1.08$ | 8.65±0.59& | 12.27±1.40€ | 0.003** |
| IL-4 | 2.67±0.17 | 1.95±0.20✠ | 2.31±0.34 | 4.25±0.10& | 5.24±0.59€ | 0.003** |
| IL-5 | 16.15±1.05 | 9.40±0.98✠ | 8.55±0.92$ | 16.58±1.89 | 28.58±2.02€ | 0.002** |
| IL-9 | 59.19±2.43 | 35.26±3.81✠ | 27.80±3.83$ | 62.59±0.78 | 105.32±9.90€ | 0.002** |
| IL-13 | 43.62±3.77 | 27.82±4.96✠ | 20.10±4.40$ | 48.19±5.51 | 83.38±7.56€ | 0.003** |

* Stands for a result of five groups comparison; ✠ represent the control group versus DSS group; $ represent the control group versus DSS+SF group; & represent the control group versus DSS+SF+EA group; € represent the control group versus SF group; * ✠ $&€, P < 0.05; **, P < 0.01. Data were presented as mean ± SEM of four mice in each group. DSS, dextran sodium sulfate; SF, sleep fragmentation; EA, electroacupuncture.

**Table S5.** Between-group comparisons of results from the multiplex immunoassay in colonic tissue in dextran sulfate sodium (DSS)-induced colitis in sleep fragmented C57BL/6 mice.

| P-value (Groups compared) | C-D✠ | C-DS$ | C-DSE& | C-S€ | D-DS✝ | DS-DSE# |
|---|---|---|---|---|---|---|
| IFN-γ | 0.468 | 1.000 | 0.564 | 0.021€ | 0.386 | 1.000 |
| TNF-α | 1.000 | 0.386 | 0.773 | 0.021€ | 0.773 | 0.386 |
| IL-1β | 0.021✠ | 0.021$ | 0.021& | 0.021€ | 0.021✝ | 0.386 |
| IL-6 | 0.248 | 0.021$ | 0.021& | 0.021€ | 0.149 | 0.149 |
| IL-10 | 0.021✠ | 0.021$ | 0.021& | 0.021€ | 0.564 | 0.021# |
| IL-23 | 1.000 | 1.000 | 0.773 | 0.021€ | 0.564 | 0.564 |
| IL-17A | 0.021✠ | 0.021$ | 0.021& | 0.021€ | 0.386 | 0.773 |
| IL-22 | 0.386 | 0.773 | 0.021& | 0.021€ | 0.564 | 0.021# |
| GM-CSF | 0.149 | 0.043$ | 0.043& | 0.021€ | 0.149 | 0.021# |
| IL-4 | 0.021✠ | 0.564 | 0.020& | 0.021€ | 0.386 | 0.020# |
| IL-5 | 0.021✠ | 0.021$ | 1.000 | 0.021€ | 0.386 | 0.021# |
| IL-9 | 0.021✠ | 0.021$ | 0.386 | 0.021€ | 0.248 | 0.021# |
| IL-13 | 0.043✠ | 0.021$ | 0.386 | 0.021€ | 0.386 | 0.021# |

✠ represent the control group versus DSS group; $ represent the control group versus DSS+SF group; & represent the control group versus DSS+SF+EA group; € represent the control group versus SF group; ✝ represent the DSS group versus DSS+SF group; # represent the DSS+SF group versus DSS+SF+EA group; ✠ $&€ ✝ #, P < 0.05. Data were presented as mean ± SEM of four mice in each group. C, control; D, DSS (dextran sodium sulfate); S, SF (sleep fragmentation); E, EA (electroacupuncture).

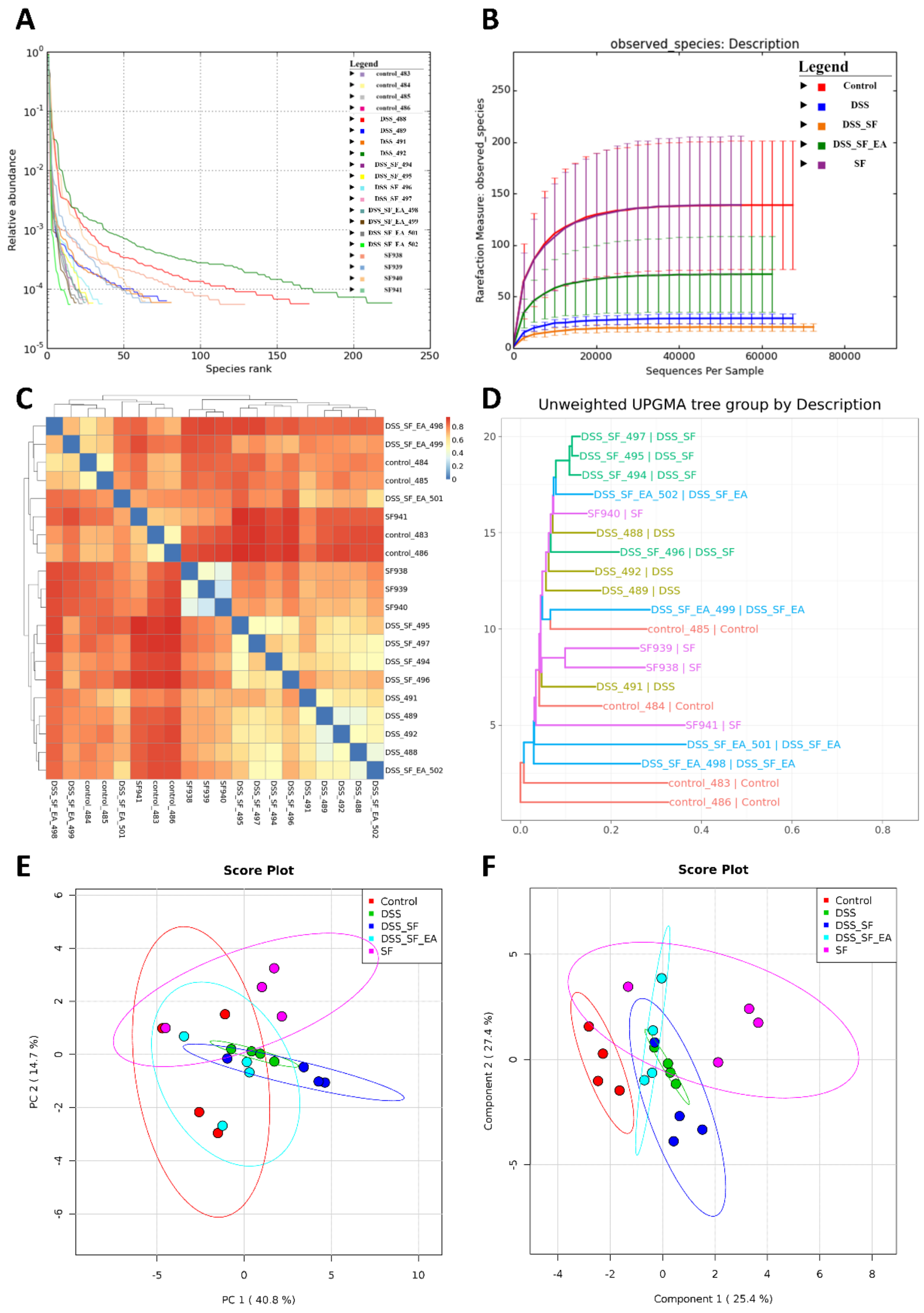
A
Relative abundance
Species rank
Legend
B
observed_species: Description
Rarefaction Measure: observed_species
Sequences Per Sample
Legend
Control
DSS
DSS_SF
DSS_SF_EA
SF
C
D
Unweighted UPGMA tree group by Description
DSS_SF_497 | DSS_SF
DSS_SF_495 | DSS_SF
DSS_SF_494 | DSS_SF
DSS_SF_EA_502 | DSS_SF_EA
SF940 | SF
DSS_488 | DSS
DSS_SF_496 | DSS_SF
DSS_492 | DSS
DSS_489 | DSS
DSS_SF_EA_499 | DSS_SF_EA
control_485 | Control
SF939 | SF
SF938 | SF
DSS_491 | DSS
control_484 | Control
SF941 | SF
DSS_SF_EA_501 | DSS_SF_EA
DSS_SF_EA_498 | DSS_SF_EA
control_483 | Control
control_486 | Control
E
Score Plot
PC 1 ( 40.8 %)
PC 2 ( 14.7 %)
Control
DSS
DSS_SF
DSS_SF_EA
SF
F
Score Plot
Component 1 ( 25.4 %)
Component 2 ( 27.4 %)
Control
DSS
DSS_SF
DSS_SF_EA
SF

**Figure S2.** EA maintain the species abundance and modulate the overall structure of gut microbiota in DSS-colitis mice with sleep fragmentation. (**A**) Rank abundance curves. Each curve represents an individual sample, plotted by relative abundance on the Y- axis and the abundance rank on the X- axis. (**B**) Rarefaction curves on observed species, means the number of species in the five groups. Rarefaction curves of OTUs sampling depth. Each curve represents a group. The sequences number is on the X-axis, and the observed species number is shown on the Y- axis. (**C**) Heatmap of unweighted distance metric of all samples, colored by unweighted distance. (**D**) Unweighted Pair Group Method with Arithmetic mean (UPGMA) tree, based on Unweighted Unifrac distance, colored by groups. (**E**) PCA (Principal Component Analysis) score. Each point represents a sample, plotted by a principal component on the X- axis and another principal component on the Y- axis, which was colored by group. (**F**) PLS-DA (Partial Least Squares Discriminant Analysis) score. Each point represents a sample, plotted by one dimension of PLS on the X- axis and another dimension of PLS on the Y- axis, which was colored by group. Samples of the control group, DSS group, DSS+SF group, DSS+SF+EA group, and SF group were represented either individually (colored by each sample name) or grouped (colored by each group name). (A-F, $n$ = 4 in each group). DSS, dextran sodium sulfate; SF, sleep fragmentation; EA, electroacupuncture.
